# Potential of ion mobility mass spectrometry in cellulose ether analysis: substitution pattern of hydroxyethyl celluloses

**DOI:** 10.1007/s00216-024-05224-w

**Published:** 2024-03-04

**Authors:** Petra Mischnick, Sarah Schleicher

**Affiliations:** Institute of Food Chemistry, Technische Universität Braunschweig, Schleinitzstr. 20, 38106 Braunschweig, Germany

**Keywords:** Electrospray ionization time-of-flight mass spectrometry, Trapped ion mobility spectrometry, Hydroxyethyl(methyl)cellulose, Tandem hydroxyalkylation versus Glc-substitution, Fingerprint of substitution pattern

## Abstract

Ion mobility mass spectrometry (ESI-tims-ToF–MS, syringe pump infusion) has been applied to glucose and oligosaccharide ethers derived from hydroxyethyl-methyl celluloses (HEMC) and hydroxyethyl celluloses (HEC) after permethylation and partial depolymerization: by hydrolysis without or with subsequent reductive amination with *m*-amino benzoic acid (*m*ABA) or by reductive cleavage. As model compounds without tandem substitution methoxyethylated methylcellulose was used. Regioisomeric glucose ethers were separated according to their ion mobility, and positions of substitution could be assigned. Glucose ethers including isomers with tandem substitution showed additional signals with a smaller collision cross-section (CCS) than core-substituted isomers. Positional isomers of cellobiose ethers were only partly resolved due to too high complexity but showed a characteristic fingerprint that might allow classifying samples. Relative intensities of signals of glucose ether isomers could only be quantified in case of ABA derivatives with its fixed charge, while sodium adducts of methoxyethyl ethers showed an influence of the MeOEt position on ion yield. Results were in very good agreement with reference analysis. [M + Na]^+^ adducts of α- and β-anomers of glucose derivatives were separated in IM, complicating position assignment. This could be overcome by reductive cleavage of the permethylated HE(M)C yielding 1,5-anhydroglucitol-terminated oligosaccharides, showing the best resolved fingerprints of the cellobiose ethers of a particular cellulose ether. With this first application of ion mobility MS to the analysis of complex cellulose ethers, the promising potential of this additional separation dimension in mass spectrometry is demonstrated and discussed.

**Graphical abstract:**

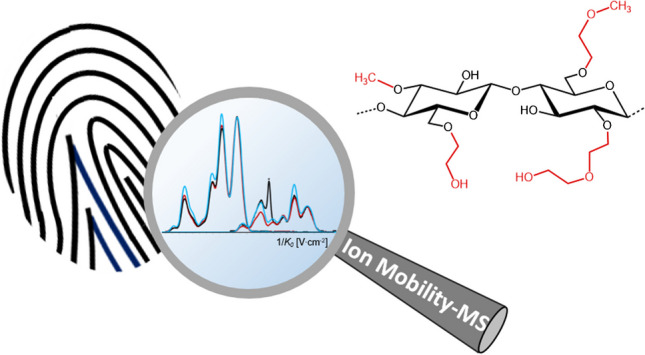

**Supplementary information:**

The online version contains supplementary material available at 10.1007/s00216-024-05224-w.

## Introduction

**Fig. 1:**
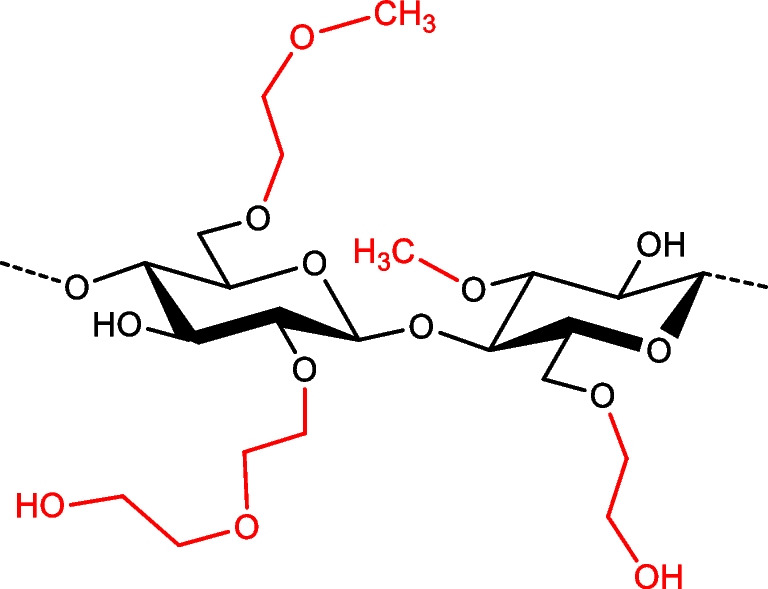
Exemplary section of the structure of hydroxyethyl(methyl)cellulose; 2,2-tandem substitution is shown at O-2’

Cellulose ethers are a versatile class of materials with a wide range of applications as additives in various industries [1], including pharma [2], food, cosmetics, and the construction sector. Their unique properties, such as water solubility, thickening, gelling, and film-forming abilities, make them indispensable in numerous formulations and products. However, understanding the structure and behavior of cellulose ethers at the molecular level remains a major challenge [3]. Among these materials, the hydroxyalkyl ethers are the most complex ones (Fig. 1) [4]. They are formed by alkali-catalyzed addition to oxiranes. Beside the three positions in the glucosyl units 2, 3, and 6, propagation to oligoether side chains is possible by consecutive reactions of the newly formed primary OH of the hydroxyalkyl function. While the degree of substitution (DS) of cellulose is limited to 3, i.e., the maximum number of OH per glucosyl unit that can be substituted, the molar degree of substitution (MS or MDS) is additionally defined as the number of added oxirane per glucosyl unit. The MDS is theoretically infinite.

Hydroxyethyl (HEC) [5, 6] and hydroxypropyl ethers (HPC) [7] are the most prominent representatives of this group. They often have MDS values > 3 or even > 4. Furthermore, mixed ethers with methyl and/or ethyl exist. These HEMC [8, 9], MEHEC, EHEC [10], and HPMC [11, 12] are characterized by comparably low MDS values, but tandem reaction is already observed at MDS as low as 0.17 [9, 11, 12]. Their characteristics depend on the molar mass and the type of substituents, as well as their DS and MDS values, respectively. Beyond the average DS and MDS, the distribution of substituents on positions 2, 3, and 6 in the glucosyl units — the regioselectivity of the reaction — and the ratio of MDS to DS, i.e., the relative portions of regio- and further constitutional isomers, as well as the distribution on the macromolecular level, have a significant impact on their properties [1, 13]. For instance, the stability of HEC of the same MDS, used as a thickener in dispersion paints, depends on the substituent distribution. Another example is the dissolution behavior of hydroxyalkyl methylcelluloses which has an impact on the pharmacokinetics when used as excipient for tablets.

Mass spectrometry is one of the most important methods in the field of the characterization of these complex materials [14, 15]. It allows to determine the substituent distribution in oligosaccharides (OS) and thus to recognize type and extent of deviations from an ideal random distribution in and over the polysaccharide chains [3, 4, 16]. We have reported on a comprehensive analysis of HEC [6] and HEMC by ESI-IT-MS [9] and MALDI-ToF–MS, earlier [8], according to the following principle: The cellulose ethers are permethylated and depolymerized by partial hydrolysis. The cellooligosaccharide (COS) mixture obtained is labeled at the reducing end with a charged tag. Each group of COS with a particular degree of polymerization (DP) is analyzed according to its number-of-substituent distribution, i.e., its MDS distribution/DP. This analysis is a relative quantification over a wide mass range (∆ *m/z* = 3∙44∙DP for HE(M)C), which was proved to be feasible with proper accuracy by a systematic evaluation of the instrumental MS measurement settings, recently [17]. However, this type of MS analysis does not differentiate between the positional isomers present for each COS of a particular DP and the number of *O*-methylated HE groups (MeOEt). Only for deuteromethylated methyl cellulose (MC), MS with collision-induced dissociation (CID) enabled to distinguish and quantify the regioisomers and thus all eight glucosyl constituents of MC from the cellobiose ether fragmentation [18]. At present, the information on the distribution of HE in the glucosyl unit can best be obtained after full depolymerization and preparation of glucitol [5, 8] or 1,5-anhydroglucitol acetates [19] by GLC-MS. However, the increasing number of possible patterns with increasing MDS does no longer provide complete resolution and structure assignment by GLC-MS. Furthermore, due to the decreasing portions of the growing number of minor components, those with the highest MDS are preferentially not detected resulting in an underestimation of the MDS.

Obviously, the analysis of cellulose substitution patterns has profited from the tremendous progress in mass spectrometry (MS) during the last decades. Electrospray ionization (ESI) and matrix-assisted laser desorption ionization (MALDI) made the formation of intact molecular ions of larger carbohydrate derivatives possible. Enhanced sensitivity and resolution helped to recognize and avoid any interference from isotopologs of related adduct ions. MS separates ions according to *m/z*, more simplified according to mass (for ions of the same charge state). By coupling with chromatographic (GLC, LC) or capillary electrophoretic separation (CE), samples can be fractionated prior to MS resulting in less interference or ion suppression of analytes. By hyphenation with the emerging technique of ion mobility spectrometry (IMS), a further dimension of separation has been added to mass spectrometry, operating in milliseconds, rather than in seconds as in chromatography. Various principles, which distinguish ions according to their collision cross-section (CCS), i.e., their size, shape, mass, and charge, more precisely, the integral of momentum transfers over all collisions with a drift gas [20], have been developed. This is especially valuable for isomeric and isobaric ions, which are not separated by the mass analyzer. The direct measure is usually the drift time of a particular ion, which depends on various parameters as the type of the collision gas, its temperature, and pressure. Drift tube (DTIMS), traveling wave (TWIMS), differential mobility analyzer (DMA), field asymmetric waveform (FAIMS), and trapped ion mobility spectrometry (TIMS) have been described and discussed elsewhere [20]. Only the principle of TIMS, applied in this study (ESI-tims-ToF-MS, Bruker), shall be briefly outlined here. In the separation funnel, incoming ions are transported by a buffer gas (nitrogen), but decelerated and — depending on their friction — finally stabilized at a certain position by an opposed electric field gradient; i.e., they are trapped in the funnel. By lowering the electric field gradient progressively, the ions are sequentially eluted in the order of decreasing *m/z* and thus decreasing CCS. Due to this principle, resolution can be significantly enhanced by choosing a shallower gradient according to a smaller range of ion mobilities. The instrument records the reciprocal reduced (regarding temperature and pressure) ion mobility 1/*K*_*0*_ in Vs∙cm^−2^. The CCS in Å^2^ is derived from *K*_*0*_ [20]. It should be mentioned here that the term collision cross-section is misleading since it is associated with the size and shape of a hard sphere. However, CCS is more related to the 3D electronic structure of the ion as a result from the positions of the atoms. Physically, it is more precisely described as the integral of the momentum transfers over all collisions [20]. Thus, it does not directly reflect the size of the ion but depends on its interaction with the drift gas, which is part of the collisions, and consequently momentum transfers are considered. Furthermore, the polarizability of analyte and drift gas has an impact on the mobilities. Due to these basics, CCS and 1/*K*_*0*_, respectively, are not simply predictable or interpretable.

IMS-MS can be coupled with CID and other fragmentation techniques (e.g., electron excitation dissociation, EED) [21] or spectroscopic methods (e.g., IR) [22] in order to gain additional structural information. It is mainly applied to biomolecules, especially in proteomics. In the field of carbohydrates, IMS-MS is a valuable tool to distinguish isomeric oligosaccharides according to their glycosidic linkage position and stereochemistry (axial, equatorial) [21–23]. Basic explorations have shown that resolution of regio- and stereoisomers also depends on the type of ion generated: protonated, deprotonated, or adducts with cations and anions, respectively. Xie et al. studied simple OS, for instance, malto- (α-1,4) and isomalto- (α-1,6) and cellooligosaccharides (β-1,4) by gated(G)-TIMS-MS and found the best resolution for netto-singly charged adducts with Ca^2+^ and I^−^ [24]. Even though deprotonation does not affect the size, the position of charge can vary due to many comparable hydroxyl functions [25]. On the other hand, cation complexation has an impact on the conformation of the carbohydrate in order to achieve the optimal number of cooperative weak interactions [25, 26]. Another problem arises from the formation of α- and β-anomer ions in the gas phase and of various sufficiently stable conformers of a distinct OS [27], consequently more than one signal per uniform compound. However, observations made for particular compounds cannot be generalized. For instance, reduction of the reducing end of some glycans, performed in order to eliminate the peak splitting due to the occurrence of anomers, does not necessarily remove the conformational heterogeneity [21]. Many of these exploratory studies deal with commercially available simple OS or glycans released from proteins, e.g., ovalbumin, or with the well-known milk OS. To the best of our knowledge, IM-MS has not been applied to the analysis of substitution patterns in polysaccharide ethers such as HEC and HEMC so far.

Therefore, we wondered whether an additional and improved insight into the regioselectivity and MDS/DS ratio would be available by measuring the COS mixtures with IMS-MS. Beside the additional information on arrival time distributions in COS, the measurement is much faster compared to the GLC method [28], and quantification does not suffer from mass fractionation effects. Here, we report on our comprehensive studies on the application of ESI-tims-ToF–MS (Bruker) for the analysis of hydroxyethyl cellulose ethers.

## Materials and methods

### Materials

#### Sample materials 

The sample materials were as follows: standards of uniform *O*-methyl-*O*-methoxyethyl-glucose and COS (2,3-*O*-methyl-6-*O*-methoxyethyl-, 2,3-*O*-methoxyethyl-6-*O*-methyl-, permethylated, and permethoxyethylated MC1 and 2 (DS_Me_ 1.29 and 1.96, respectively) [17], permethylated HEMC1-3 (MDS_Zeisel_ 0.21, 0.35, and 0.17, respectively) [9], and permethylated HEC1–3 (MDS_Zeisel_ 1.85, 2.06, and 3.35, respectively) [6]. The Zeisel value is determined by the producer to specify the material according to its MDS. It is a modified method, going back to the cleavage of cellulose ethers by the strong acid HI and the quantification of the formed cleavage products.

#### Chemicals

With the exception of DMSO (≥ 99.5% for synthesis) and trifluoroacetic acid (TFA) (≥ 99.9%), purchased from Roth, as well as acetic acid (≤ 99.8%, LC–MS quality) (HOAc) from VWR, all chemicals were purchased from Sigma-Aldrich/Merck and were of p.a. grade. For ESI–MS measurements, solvents of LC–MS grade were used.

#### Derivatives

The derivatives were as follows: α,β-mono- and cellooligosaccharides (α,β-COS), 1,5-anhydroglucitol-terminated COS (COS-red), and *m*ABA-labeled COS (COS-ABA). α,β-COS were obtained by partial acid hydrolysis and COS-ABA by subsequent reductive amination with *m*-amino-benzoic acid (*m*ABA) as described earlier [9].

COS-red were obtained by partial depolymerization using reductive cleavage [19, 29]: 1–2 mg of the peralkylated cellulose ether is dissolved in dry dichloromethane in a screw-cap vial. Per glucosyl unit, 3 equiv. triethylsilane, 3 equiv. TMS mesylate, and 0.6 equiv. BF_3_∙OEt_2_ are added. After stirring for 2 h at r.t., the reaction is quenched with saturated NaHCO_3_ solution. The organic phase is washed with water several times and dried over CaCl_2_. For ESI–MS, the sample is diluted with methanol. Concentrations of MS-samples were between 0.005 and 0.02 mg/mL.

### Instrumentation

Ion mobility studies were performed on an electrospray ionization-trapped ion mobility spectrometry-time of flight mass spectrometer (ESI-tims-ToF–MS, version tims-ToF classic from 2021, Bruker Daltonics, Bremen, Germany). The samples were measured in detect or ultra-mode and applied by syringe pump infusion (180 μL/ h). The detect mode allows mobility measurement over a wide mobility range (∆ 1/*K*_*0*_ = 1.24) and thus also mass range, however with a lower resolution in the range 50–90. A higher resolution (110–190) is achieved in the ultra-mode, but the mobility range is limited to ∆ 1/*K*_*0*_ = 0.3, so that the mass range to be analyzed had to be divided into smaller measurement segments with different mobility windows, depending on the sample and derivative type. Each measurement segment was recorded for 3–5 min. Furthermore, in order to recalibrate the data after the measurement, a calibration segment was set in each measurement, in which the calibration solution was infused for 0.5–1.5 min. An accumulation time of 20 ms and a rolling average of 5 were used.

The measurements were carried out in positive or negative mode, respectively, depending on the derivative type of the sample. The ion source parameters were as follows: Nitrogen was used as dry gas (4 L min^−1^, 220 °C); nebulizer gas (1 bar); capillary 4.0 kV; and end plate offset 500 V. Tune parameters were as follows: deflection 1, Δ 80 V, funnel 1 RF 500 Vpp, funnel 2 RF 250 Vpp, multipole RF 200 Vpp, in source collision-induced dissociation (isCID) energy 0 eV, ion energy 5 eV, collision energy 7 eV, collision RF 700 Vpp (DP1)/1000 Vpp (≥ DP2), transfer time 65 μs, and pre-pulse storage time 5 μs. Mass spectra were recorded from *m/z* 100 to 1600.

## Results and discussion

For the ion mobility studies of hydroxyethyl derivatives of celluloses, permethylated MC, HEMC, and HEC as well as permethoxyethylated MC were partially depolymerized. Furthermore, some regioselectively etherified standards were included in order to assign the signals in the ion mobilograms (IM) regarding the position of substitution. Beside the α,β-COS, 1,5-anhydroalditol-terminated COS (COS-red) were measured, both as sodium adducts in positive mode, further *m*ABA-labeled COS (COS-ABA), obtained by reductive amination with (*m*ABA), in negative mode, respectively. All samples were alkylated in positons 2, 3, and 6. Sample solutions were applied by syringe pump infusion.

### ***Permethylated α,β-COS***

First, a hydrolysate of permethylated MC was measured for a first orientation of the ion mobilities of these COS, given as 1/*K*_*0*_. While the ion mobility decreases with the molar mass, reciprocal 1/*K*_*0*_ correlates with the collision cross-section (CCS in Å^2^), which are thus derived from the measured ion mobilities, taking into account the reduced mass of drift gas and analyte and the charge state [20]. As described above, tims is a trap-and-release technique. Resolution depends on the field gradient applied. Thus, in a smaller window of mobilities, higher resolution is achievable. For a first orientation, the detect mode was applied, which allows the measurement of a larger range of ion mobilities (here, 1/*K*_*0*_ 0.6–1.8). Subsequently, the higher resolving ultra-mode was applied. This mode requires 3–4 measurements of a HE-COS sample with settings for smaller mobility segments of about Δ 0.3 for 1/*K*_*0*_. (Unless otherwise stated, the results obtained from the ultra-mode measurements are given in the following.) In both modes, 2,3,6-tri-*O*-methyl-α,β-d-glucose (α,β-COS, DP1, [M + Na]^+^ *m/z* 245) showed two signals at 1/*K*_*0*_ 0.707 ± 0.002 and 0.721 ± 0.002 (detect mode, *n* = 6). In the detect mode, α,β-COS of DP2 and DP3 gave only one signal. When each DP was measured in the ultra-mode under conditions for the corresponding mobility window, two isomers could be observed for all COS, of which DP3 was only partially separated.

The detection of two species per DP is attributed to the α- and β-anomer of glucose or the glucosyl unit at the reducing end of α,β-COS, respectively, since after reduction, only one signal is observed. Monosaccharides are relatively rigid, and by metal cation coordination, their conformation is additionally fixed. Sodium complexation of the monosaccharide probably involves the hemiacetal OH at C-1 (more acidic, higher electron density) and OCH_3_ at C-2. In the α-anomer, these functional groups are in *cis-*, in the β-anomer, they are in *trans*-relation. The separation of anomers has been described by several authors [30–32]. Dwivedi et al. [30] separated the sodium adducts of α- and β-methyl glycosides of some hexopyranoses (Glc, Gal, Man) by drift time IMS in N_2_. The β-anomer was always eluted first, and the order of the sugars with respect to drift time and thus CCS was Man < Gal < Glc. Warnke et al. [31] studied sodium adducts of glucose. They monitored the anomerization, starting with α-Glc and observed an increasing signal of “smaller” β-Glc (at shorter drift time). This example shows that the shape and CCS of ions are not simply predictable. Beside size and surface, polarity also has an impact and can cause various IM at the same shape and size of the ion [20, 33]. Equatorial OH in pyranoses exhibit stronger field effects than the axial ones [34]. Molecular dynamics calculations of [M + Li + H_2_O]^+^ of α-Me-Man and α-Me-Glc suggested that in Glc, O*-*3, O-4, and O-6 are involved, whereas its 2-epimer used the axial O-2, ring-O-5, and the more flexible primary O-6 for Li^+^ coordination [35]. With the first glycosidic linkage in cellobiose, conformational freedom is enhanced, since the two glycosyl units can freely rotate around the C1-O-C4‘-linkages. Now, Na complexation is energetically more favored between the two sugar units, allowing a higher *O*-coordination number [26, 36], the optimum of which is 6 for Na^+^. Since after reduction (COS-red, COS-ABA, see below) only one signal is detected for each DP with uniform pattern, it is obvious that the appearance of two separated signals is attributed to the existence of two stereoisomers.

### O-***Methoxyethyl***-O-***methyl COS***

After this first orientation, two methoxyethylated MC [17] were studied (MC1 DS_Me_ 1.29, MC2 DS_Me_ 1.96). These compounds represent permethylated HEMC or HEC but without tandem substitution. Thus, MDS_HE_ = DS_HE_ = 3.0 − DS_Me_. The methoxyethyl (MeOEt = methylated HE) pattern is complementary to the methyl pattern, and the general regioselectivity is inverse (3 > 6 > 2) and known in detail from extensive analysis of the original MC. In addition, regioselectively substituted standard compounds, 2,3-di-*O*-Me-6-*O*-MeOEt- and 2,3-di-*O*-MeOEt-6-*O*-Me- [17], were used to confirm signal assignments and aid in pattern evaluation. The tri-*O*-alkylated α,β-glucose standards all showed two signals of comparable intensity, except the 2,3-di-*O*-MeOEt-6-*O*-Me-Glc, showing only a single signal. This compound most likely binds the cation between the two neighboring substituents, which can coordinate the sodium ion with four oxygens, flexible enough to assume a position in optimal distance to the cation. In case of tri-*O*-MeOEt-Glc, this comfortable coordination is also possible. However, the additional MeOEt in the primary position affects the sodium complexation properties, resulting in two isomeric ions of different CCS, possibly attributed to the α- and β-anomer or to another sufficiently stable sodium adduct involving the crown ether-like MeOEt ensemble.

**Fig. 2:**
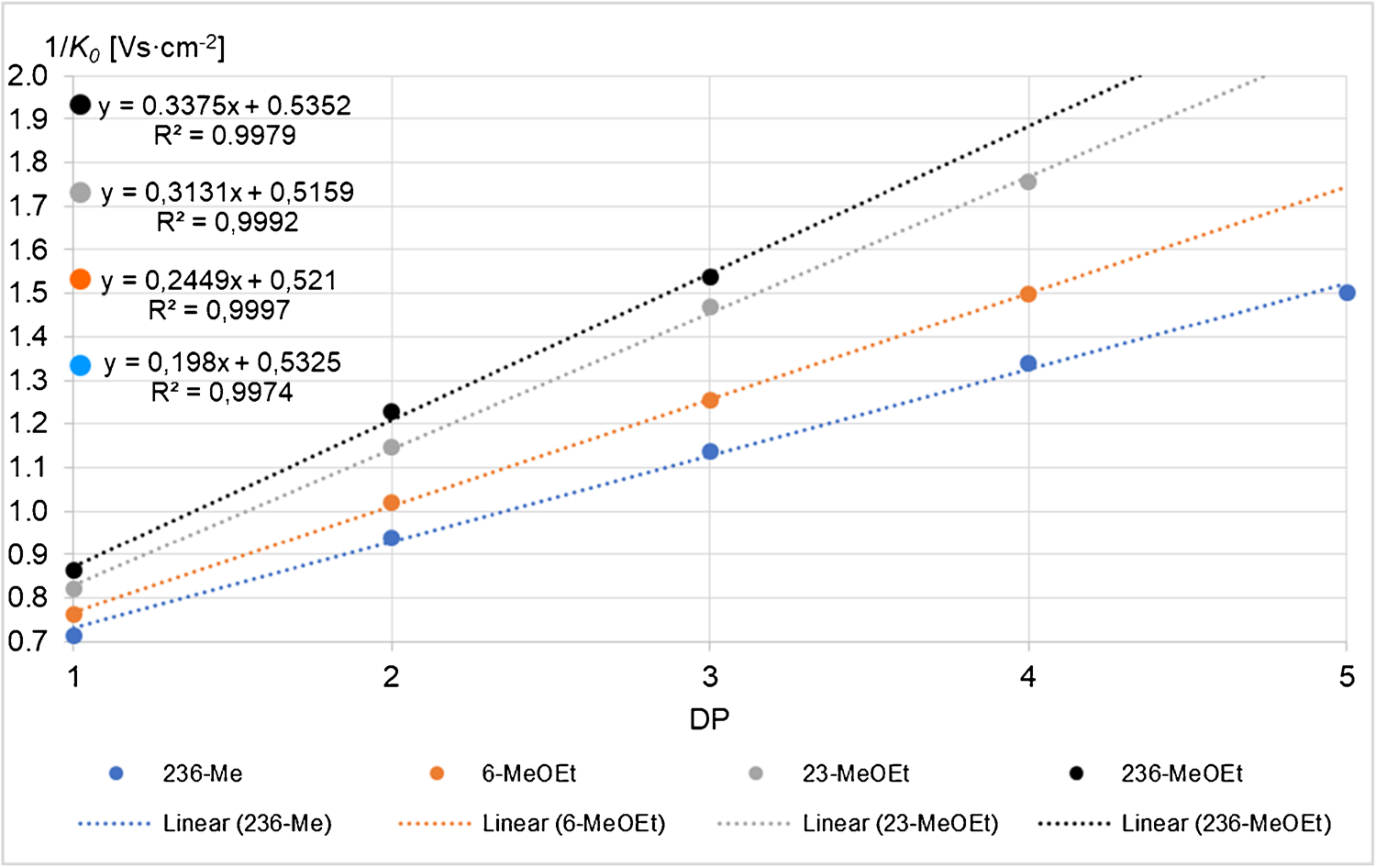
Reciprocal reduced ion mobility 1/*K*_*0*_ in VS∙cm^−2^ of homologous α,β-COS, [M + Na]^+^, with increasing number of MeOEt/Glc, n(MeOEt) + n(Me)/Glc = 3, *m/z* 245–1061. ESI-tims-ToF–MS, positive, detect mode, syringe infusion. Average values of α- and β-anomers are shown if separated; in detect mode, not all α- and β-COS were separated. Linear fits are shown. For a respective diagram displaying 1/*K*_*0*_ against *m/z*, see ESM Fig. S1a. All 1/*K*_*0*_ values measured for 236-Me-α,β-COS, DP1-5, are listed in the ESM, section 1. Standard deviations (SD) are in the range of ± 0.18 to ± 0.30%. For 236-MeOEt-α,β-COS SD for DP1-3 (*n* = 3) was ± 0.27%, ± 0.45%, and ± 0.61%, respectively

Figure 2 shows the increase of the reciprocal reduced ion mobilities 1/*K*_*0*_, briefly ion mobilities (IM), of α,β-COS with the DP for all patterns: 2,3,6-Me, 2,3-Me-6-MeOEt, 2,3-MeOEt-6-Me, and 2,3,6-MeOEt. The average value of α- and β-anomers is given. An additional graphic relating 1/*K*_*0*_ to *m/z* and further information are given in the ESM, Fig. S1a,b. The 1/*K*_*0*_ values increased approximately linearly with the number of MeOEt/Glc within one DP. A polynomic regression fits slightly better, but for the range under consideration, the difference is small. Looking into the development of 1/*K*_*0*_ with n(MeOEt) for each DP (Fig. S1b), it is obvious that the increase from no to 6-MeOEt is significantly smaller than from 6- to 2,3-MeOEt. This reflects already the influence of the position with the adjacent MeOEt having a stronger impact on the mobility.

From comparison of the IM of MeOEt-MC and of the α,β-COS standards, it can be deduced that the sodium adduct of 6-*O*-MeOEt can adapt the most compact shape of the mono-methoxyethylated regioisomers. On the contrary, the 2,3-di-*O*-MeOEt substitution pattern induces the largest CCS among the di-methoxyethylated isomers. However, full resolution and signal assignment of isomers in mixtures from real samples are not possible due to the appearance of the α,β-anomer pairs for several patterns of DP1. Furthermore, a change of the anomer ratio with the accumulation time has been observed, indicating that the dynamic of anomerization might cause inconsistencies of IM profiles.

### ***1,5-Anhydro-glucitol-terminated COS (COS-red)***

In order to reduce the complexity and to ensure a stable sample composition over time, we decided to eliminate the formation of anomeric Na complexes by reduction of the terminal carbonyl function (“reducing end”). Since we expected a better differentiation of regioisomers when the substituents are in a fixed orientation in a cyclic pyrane structure, we performed a partial reductive depolymerization [19, 29], promoted by a Lewis acid and in situ reduction of the oxocarbenium ion formed by triethylsilane, yielding 1,5-anhydro-glucitols (DP1) and 1,5-anhydro-glucitol-terminated COS (COS-red). Furthermore, this procedure yields the reduced analytes in one step at room temperature, while reduction with NaBH_4_ after hydrolysis would require a second step with more tedious work-up, and contaminate the sample with salt. Molar masses of COS-red are 16 Da less than for α,β-COS. Again [M + Na]^+^ ions were measured.

The IMS of DP1 and 2 of MC-MeOEt showed a reduced number of signals. For the uniformly substituted standard compounds, only one peak was observed now (except a minor side peak for 236-Me for DP ≥ 2, less than 6%). 1/*K*_*0*_ of COS-red is comparable to the β-COS anomer.

**Fig. 3:**
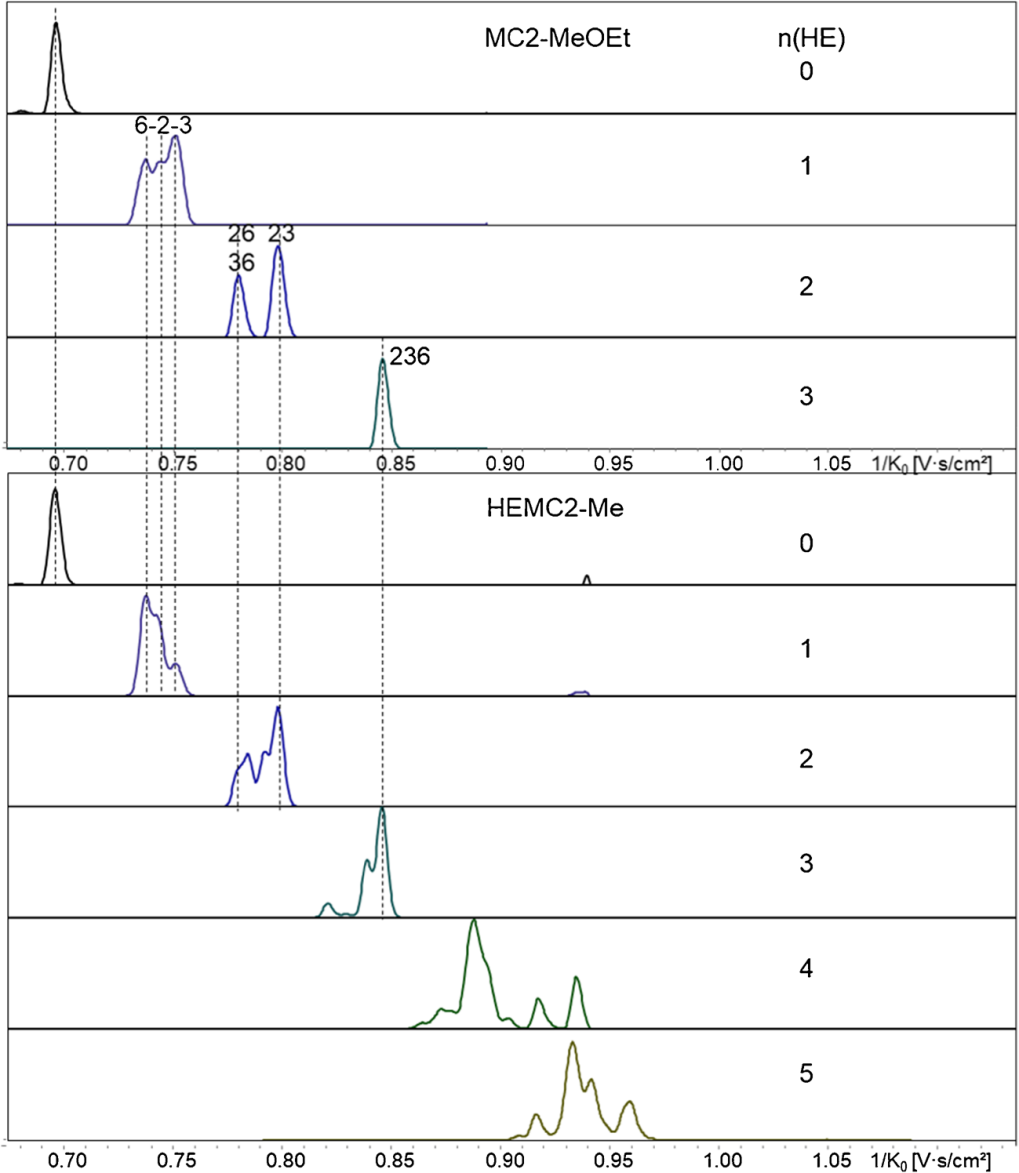
EIM list of MC2-MeOEt (MDS_HE_ = DS_HE_ = 1.04), COS-red, DP1, *m/z* 229–361 (top), and of HEMC2 (MDS_HE_ 0.35), COS-red, DP1, *m/z* 229–449 (bottom). [M + Na].^+^, ESI-tims-ToF–MS, positive, ultra-mode, syringe infusion. Signals of direct Glc-substitution (no tandem) are assigned according the position of MeOEt (= methylated HE) and are correlated with the more complex profiles of the HEMC (MS 0.35). All additional peaks in HEMC result from tandem substitution reaction, starting with n(HE) = 2. Signal intensities of various EIM cannot be compared since all were normalized to their intensity maximum. EIM of HEMC, n(HE) = 4 and 5, are smoothed. For relative intensities, see ESM, Fig. S2

For monosubstituted 1,5-anhydro-glucitols (COS-red, DP1), the three, not fully separated peaks in the IM could now be assigned to the three regioisomers. With the help of the corresponding standard derivative and the GLC reference data (MC2-MeOEt, 10% in 2-, 68% in 3, 21% in 6-position), the order regarding the reciprocal mobilities 1/*K*_*0*_ can be confirmed to be 6-, 2-, and 3-*O*-MeOEt (means, 6-*O*-MeOEt has the lowest CCS). For the disubstituted ones, two well separated peaks are observed, of which the one with the higher 1/*K*_*0*_ agrees with the 1,5-anhydro-2,3-di-*O*-MeOEt-6-*O*-Me-glucitol standard. Thus, the first peak is attributed to the more similar 2,6- and 3,6-MeOEt-isomers. Figure 3 (top) shows the extracted ion mobilograms (EIM) of the methoxyethylated MC2 (COS-red, DP1).

### ***Tandem substitution***

Comparison of the MC-MeOEt with permethylated HEMC and HEC (HEMC-Me, HEC-Me) allows to identify the additional signals of DP1 caused by tandem substitution, starting with n(HE) = 2 (Fig. 3, bottom). From the reference data obtained by GLC-FID [9], it is known that the main patterns in the disubstituted fraction of HEMC2 are 6,6-, 2,2-, and 2,6-HE (all around 30%), while 2,3-, 3,6-, and 3,3-HE are minor constituents. Compared to the methoxyethylated MC, the additional signals related to tandem substitution are located between the other isomers but also overlap (Fig. 3). The relative signal abundance at 1/*K*_*0*_ of 2,3-HE indicates that there must be another pattern behind, probably 2,2, since the order of the tandem products is expected to follow that observed for monosubstituted units: thus, 6,6–2,2–3,3. In GLC analysis of these low-MDS HEMCs, not even the 2,3,6-tri-*O*-HEme glucitol has been detected. On the contrary, in ESI–MS, up to pentasubstituted Glc comprising several isomers are visible for HEMC2 (MDS 0.35) (Fig. 3). For n(HE) ≥ 4, however, the absolute intensity becomes marginal (Fig. S2).

**Fig. 4:**
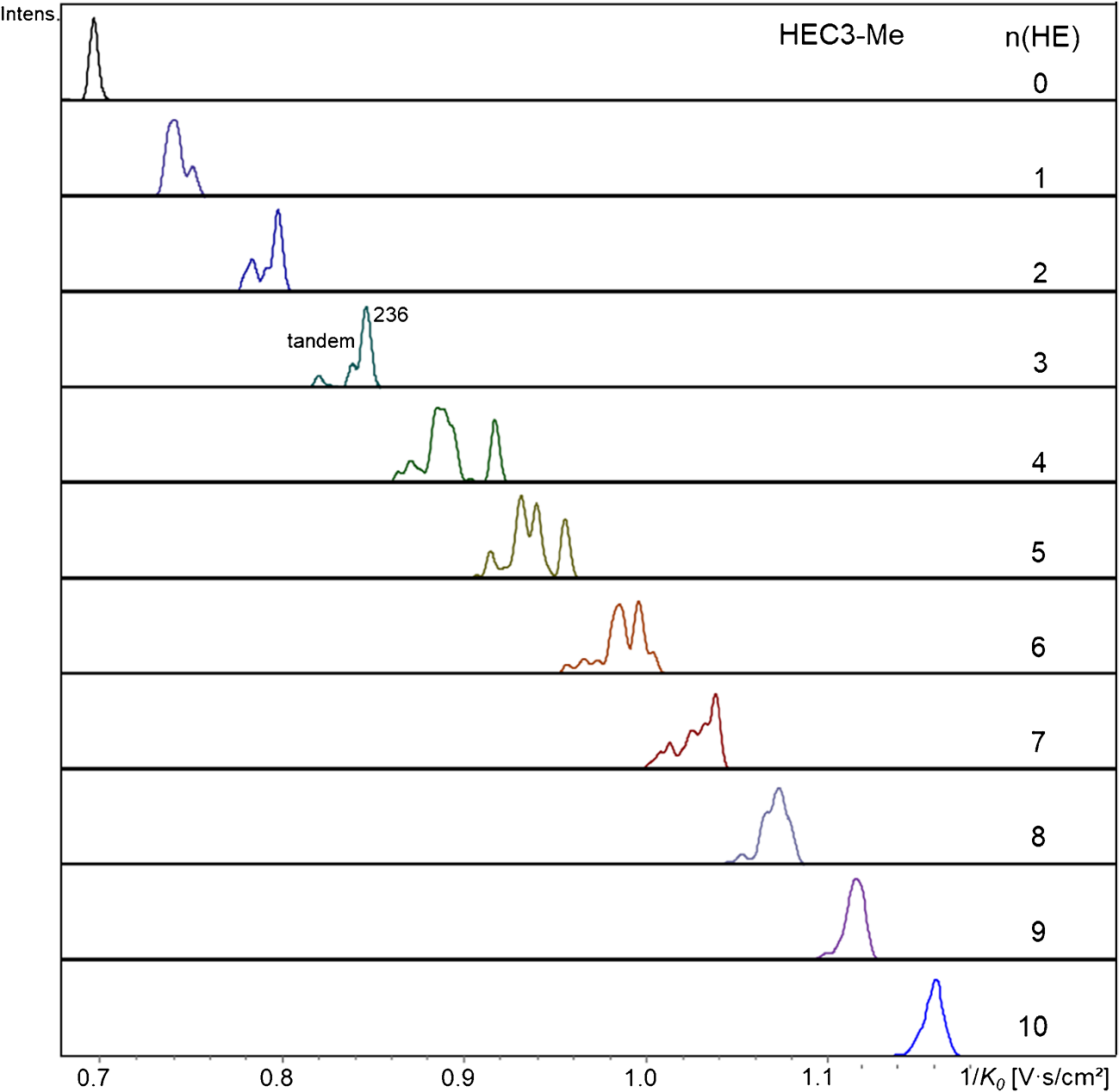
EIM list of permethylated HEC3, MDS 3.35, COS-red, DP1, n(HE) = 0–10, *m/z* 229–669, [M + Na].^+^, ESI-tims-ToF–MS, positive, ultra-mode, syringe infusion. Please, notice the different intensity scales of the particular EIM. For comparison with HEMC-2, see ESM, Fig. S3

As an example of a sample with longer oligoether side chains, HEC3 [6] with an MS of 3.35 is shown in Fig. 4. A direct comparison of HEC3 with HEMC2 shows qualitatively the same patterns but with different relative intensities within a group of isomers (ESM, Fig. S3). Anhydro-glucitols with up to 10 HE were detected for DP1 of HEC3. The number of partially resolved isomers increased up to *n* = 7, but beyond this number, it decreased again. Probably with conformational freedom of the growing side chains, they no longer cause big differences in the overall shape and polarity.

Evaluation of the peak areas of regioisomers of COS-red of MC-MeOEt showed that these do not match the molar proportions known from GLC analysis. 2-*O*-HE is the most overestimated one, followed by 6-*O*-HEme, while 3-*O*-HEme is relatively underestimated. This indicates different ion yields, since mass fractionations does not apply, probably due to different Na complexation behavior, most pronounced for DP1. The stability of sodium complexes increases with the number of O available for complexation in an appropriate position, up to a coordination number of 6. For HE ethers, it is known that due to the substituents’ impact on sodium complexation, COS should be labeled with a charge-bearing tag for quantitative MS [37–39]. The results of IMS reveal that not only the number, but also the positions of these substituents have an impact on the relative abundance of Na complexes.

### ***mABA-labeled COS (COS-ABA)***

Therefore, in the next step, we measured the corresponding 1-(3-carboxyphenyl)-amino-1-deoxy-glucitols, obtained by reductive amination with *m*ABA. In earlier studies on substituent pattern analysis of COS ethers by MS, *m*ABA has been established as an appropriate label [12]. Like the 1,5-anhydro-alditols, these compounds can no longer form α- and β-anomers but in contrast feature an open-chain structure and consequently more conformative flexibility, while the charge position is fixed at the tag. This localization should prevent discrimination possibly caused by different sodium complex stabilities [9, 17].

**Fig. 5:**
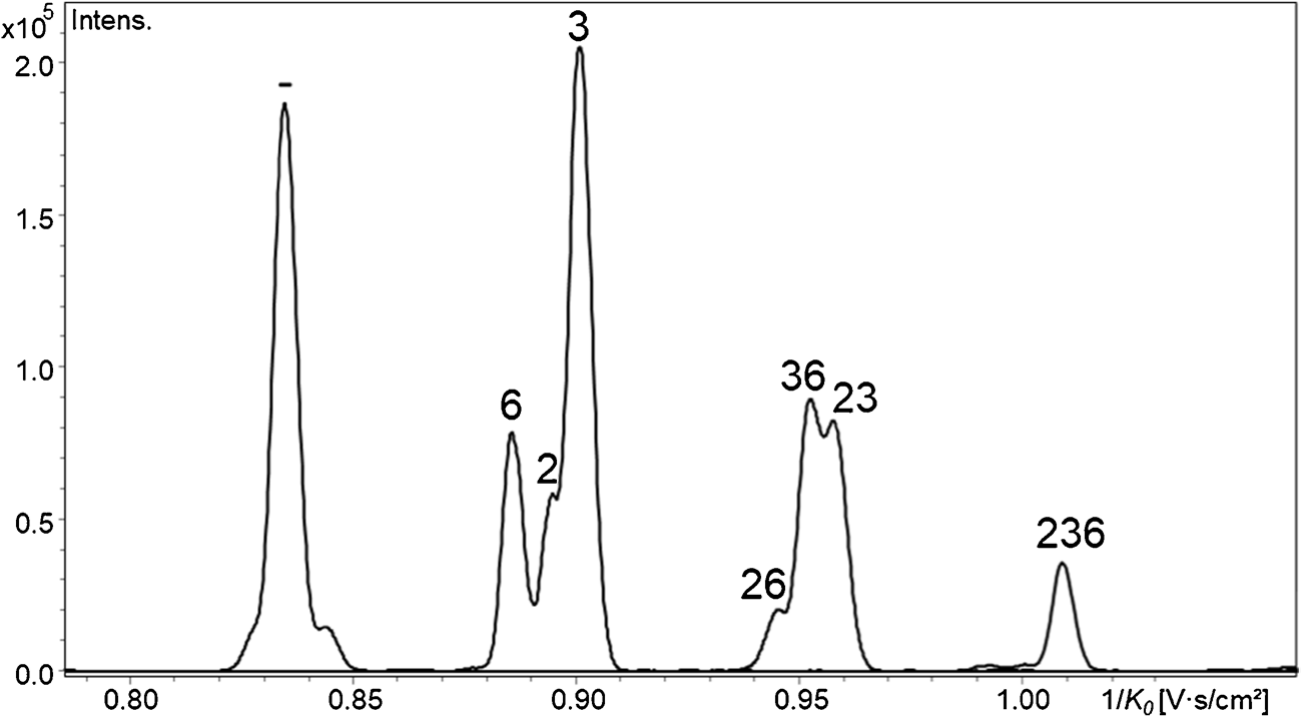
Overlaid EIM of MC2-MeOEt, ABA-glucitols (COS-ABA, DP1, *m/z* 342–474), [M-H]^−^. ESI-tims-ToF–MS, negative, ultra-mode, syringe infusion

For DP1 of COS-ABA (Fig. 5), an even better resolution of the groups of mono- and disubstituted regioisomers was obtained than for the sodium adducts of 1,5-anhydro-alditols, following the same order (regarding increasing 1/*K*_*0*_ and thus CCS): 6–2–3 and 2,6–3,6–2,3. The assignment of the 6-*O*- and the 2,3-di-*O*-methoxyethyl ethers was proved by the corresponding standard derivatives.

**Fig. 6:**
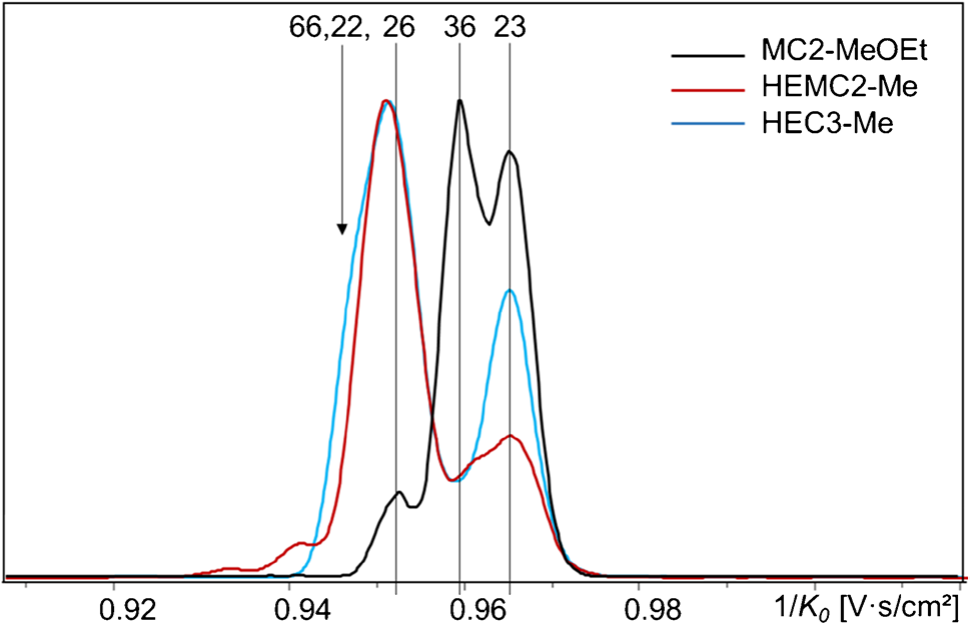
Comparison of the EIM of three HE/Me samples, COS-ABA, DP1, n(HE) = 2 (*m/z* 430). ESI-tims-ToF–MS, negative, ultra-mode, syringe infusion, [M-H]^−^. Methoxyethylated MC2 (inverse regioselectivity for HE, DS_HE_ = MDS_HE_ = 1.04, no tandem products), methylated HEMC2 (MDS_HE_ 0.35), and methylated HEC3 (MDS 3.35). Ion mobilograms are aligned according to 2,3-HE

The remaining isomers were assigned according to their very different portions in methylated HEMC and otherwise the complementary methoxyethylated MC (Fig. 6). While for the tri-*O*-methoxyethyl glucitol (Fig. 5, peak 236) only a single signal was detected, the also uniform tri-*O*-methyl-ether (Fig. 5, peak −) showed a small shoulder in front and a separated small signal behind the main peak (Fig. 5). It is difficult to appraise, whether these are artifacts or represent minor contributions by alternative stable conformers.

Integration of the IM peak areas of ABA-glucitols and normalization within the regioisomer fractions gave proportions, which were in good agreement with the complementary methyl pattern determined by GLC. Deviations are probably due to incomplete separation (Table 1).

**Table 1:** Quantitative evaluation of IM-MS of MC-MeOEt-ABA and comparison with GLC reference data. ESI-tims-ToF–MS, negative, ultra-mode, syringe infusion

Position of MeOEt	MC1-MeOEt Mol % (GLC)	Relative portions of regioisomers (GLC)	Relative portions of regioisomers (IM-MS) (*n* = 2)	MC2-MeOEt Mol % (GLC)	MC2-MeOEt Mol % (IM-MS) (*n* = 2)	Relative portions of regioisomers (GLC)	Relative portions of regioisomers (IMS) (*n* = 3)
*-*	8.9			29.3	29.1		
*2*	3.2	10.5	10.7	4.7	4.8	11.2	11.1
*3*	21.4	69.4	66.9	28.3	27.8	67.5	66.4
*6*	6.2	20.0	22.4	9.0	9.5	21.3	22.5
*23*	15.9	38.9	41.3	10.1	10.1	42.3	43.2
*26*	2.4	5.8	6.8	1.4	1.9	5.8	7.0
*36*	22.7	55.3	51.9	12.3	11.7	51.9	49.8
*236*	19.3			4.9	5.1		
DS	1.71			1.04	1.04		

Even the evaluation of the total composition, including non- and trisubstituted constituents, i.e., analytes of different *m/z*, showed a surprisingly good agreement in case of MC2-MeOEt. For MC1-MeOEt, a slight discrimination with increasing *m/z* and consequently a 5% too low DS_MeOEt_ were obtained. Mass fractionation effects are less pronounced in ToF analysis compared to ion traps, for instance. Pulse times after elution of ions from the tims device were probably sufficient for the *m/z* range of interest. It should, however, be kept in mind that the measurement conditions were not optimized in order to overcome mass selective effects but were focused on the regioisomer ratios at the time of the measurement of MC1-MeOEt. Whether quantification beyond this object of investigation is feasible would require more comprehensive studies.

As described above for COS-red, isomers from HE-chain propagation were identified by comparison of tandem-free methoxyethylated MCs with methylated HEMC and HECs. Starting with n(HE) = 2 (DP1, *m/z* 430), tandem substitution emerges. This group comprises six isomers: beside 23-, 26-, and 36-, the tandem substitution products 22-, 33-, and 66-HE. Comparison of the EIM (COS-ABA, DP1) of the three types of samples (Fig. 6) shows an additional broadened and distorted first signal, starting earlier than 26-HE; the signal of 36-HE is no longer well visible, since its contribution is only 6–8% (as known from the reference data), but it can be assigned at the broad saddle between the two bigger signals. The last peak matches the 23-HE-isomer.

From the peak areas, it can be concluded that the broadened signal comprises 66-, 22-, and 26-HE. Whether 33-HE is also part of the tandem group cannot be decided due to its small portion of less than 2% [6]. Table 2 shows the quantitative evaluation of these fractions for HEC1–3.

**Table 2:** Molar composition of the HE-disubstituted ABA-glucitol isomers (DP1, n(HE) = 2) as determined by GLC of alditol acetates [6] and ESI-tims-ToF–MS, ultra-mode, *n* = 1

Position of HE	HEC1 GLC	HEC1 IMS	HEC2 GLC	HEC2 IMS	HEC3 GLC	HEC3 IMS
*66*	8.9		12.6		15.1	
*22*	16.9		15.6		20.6	
*26*	41.2		36.7		29.9	
*66* + *22* + *26*	67.0	66.7	65.0	67.7	65.6	67.7
*36*	7.5	7.2	7.9	4.1	6.3	3.1
*23*	23.7	26.1	25.2	28.1	28.1	29.3
*33*	1.8		2.0		-	

**Fig. 7:**
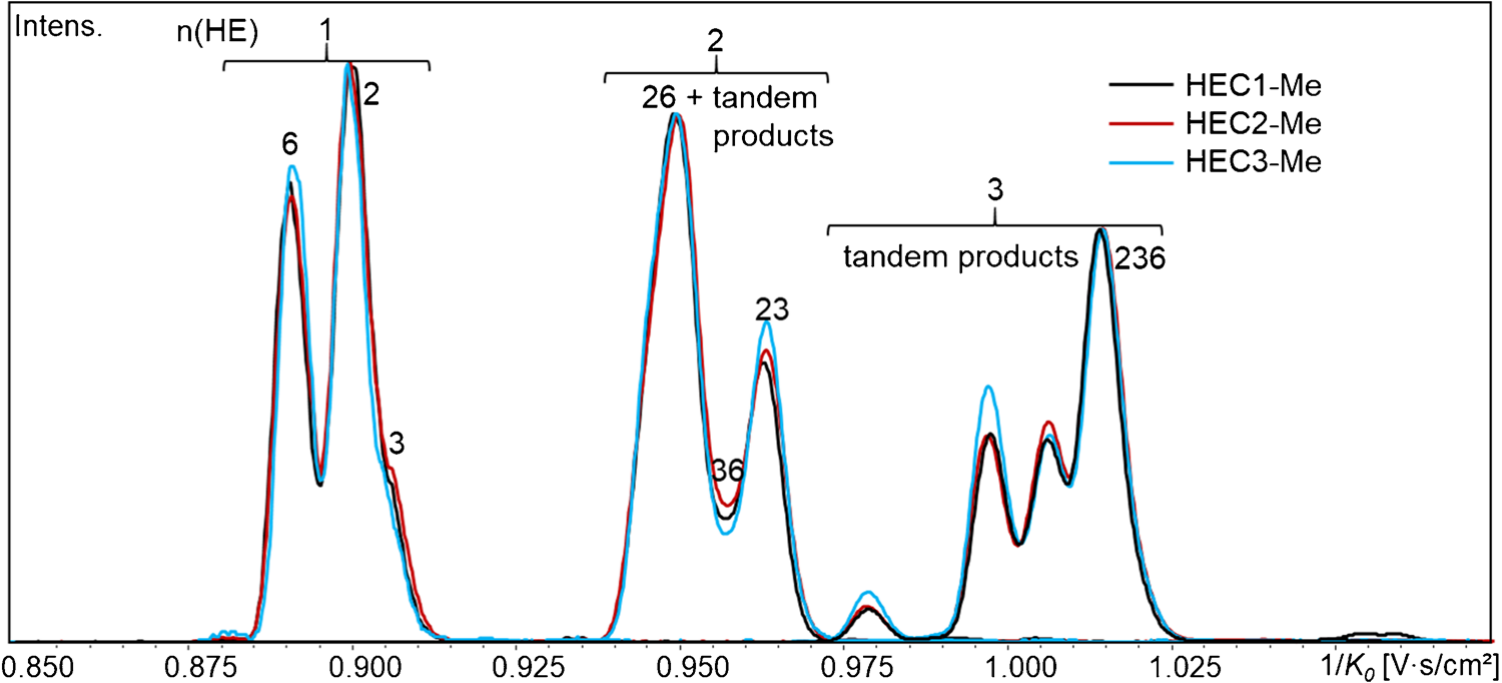
Overlaid EIM of HEC1–3-Me, COS-ABA, DP1, n(HE) = 1–3 (*m/z* 386, 430, 474). Relative intensities are normalized to the maximum signal of each EIM. The signal at 1.014 contains 2,3,6-HE plus isomers; all other signals present tandem products of the type xxy and xxx

The next group with n(HE) = 3 of ABA-labeled HEC-Me (DP1, *m/z* 474) comprises already ten isomers: beside 236 (MDS/DS = 1), the tandem products 223, 226, 233, 336, 266, and 366 (MDS/DS = 1.5) and 222, 333, and 666 (MDS/DS = 3). Except 333, all isomers have been detected by GLC-MS analysis [6]: In all HECs, 236 dominates (GLC, 37, 37, 27%), followed by 223 (+ 233, not separated), 226, and 266. Figure 7 compares the EIM for n(HE) = 1–3 of the three HEC. The signal at 1/*K*_*0*_ 1.014 can be attributed to the 2,3,6-tri-*O*-methoxyethyl glucitol, but due to its abundance, the peak should also contain one or more other isomers. Especially for HEC3 with the largest MDS, it is visible that this signal relatively decreases, while the tandem substitution products, presented by all other signals, increase. Also for HEC1 and HEC2 with more similar MDS, a deviation of their relative patterns can be observed. Further comparison of IM fingerprints for n(HE) = 4–10 is shown in the supplement material (ESM, Fig. S4a).

A comparison of the mobility range of each HEC of COS-ABA, DP1, from permethylated HEC1–3 is displayed in ESM, Fig. S4b. The increasing MDS (1.85, 2.06, and 3.35) is obvious from the shift of relative intensities to constituents with larger number of HE. The evaluation of these relative intensities, i.e., the n(HE) distribution in the HECs, yielded MDS values which are close to the Zeisel values, provided by the supplier (see ESM, Table S1). While in GLC, n(HE) = 7 was the limit of detection and the MDS underestimated, small portions of higher tandem products up to n(HE) = 10 (HEC3) were detected by IMS-MS, and a better agreement with the Zeisel value was obtained. It should be kept in mind that conditions were not optimized to exclude mass fractionation effects, and an evaluation over such a large *m/z* range is usually not applicable. However, the average MS values obtained demonstrate that this discrimination is impressingly low in the ToF–MS.

The standard deviation (SD) of 1/*K*_*0*_ was calculated for two uniform compounds, the 2,3,6-tri-*O*-methyl- and tri-*O*-methoxyethyl-1,5-anhydro-glucitols and ABA-labeled glucitols. The intraday SD of absolute 1/*K*_*0*_ values for samples measured under the same conditions was between ± 0.05 and ± 0.22 (n = 3–5). For samples measured on different days, deviation was in the range of ± 0.2% up to ± 1% and thus in the range of the differences between the ion mobilities of some isomers. However, those uncertainties were mainly caused by a systematic deviation. Thus, IM profiles of various samples can be aligned for comparison as has been shown in some figures above and in the ESM.

Naturally, the very complex mixtures from depolymerization of HE-cellulose ethers cover a wide range of concentrations of constituents, summarized in isomer fractions. With decreasing ion intensity, S/N decreases, thus limiting evaluation/interpretability, first quantitatively and finally qualitatively. Up to intensities of 10^4^, the shape can often reasonably be adjusted by smoothing (with exceptions); however, at lower concentrations, the ion mobilograms are no longer meaningful at all.

### ***COS*** ≥ ***DP2, fingerprints***

By ESI-tims-ToF–MS of partially depolymerized cellulose ethers, not only detailed information about the patterns of the glucose constituents (DP1) is obtained, but in addition IM of DP2 and larger COS can be recorded from the same sample material. In contrast to the glucose derivatives, in case of DP2, the 1,5-anhydroglucitol-terminated derivatives (COS-red) showed a better resolution for n(HE) ≥ 2 than the ABA-labeled ones; however, the maximum number of resolved signals (without tandem products) was 4–5. An overview of both types of derivatives of MC2-MeOEt is shown for DP2, n(HE) = 0–6, in the ESM (Fig. S5). These profiles can be used as a fingerprint of a particular cellulose ether and might provide additional valuable information in case of many collected data, related to physical properties. In this context, fingerprint does not refer to a particular constituent but to the cellulose ether material it is derived from. For this purpose, quantifiability is no longer that important, rather the resolution and comparability in order to enhance the informational value. Therefore, 1,5-anhydroglucitol-terminated DP2 is especially considered here. Besides, the impact of the substitution pattern on sodium complexation and thus on the ion yield is expected to be leveled with increasing number of coordination sides.

With the DP, the number of theoretical substitution patterns strongly increases. Complexity also increases with the number of substituents/DP, going through a maximum when 50% of the possible positions are occupied. For DP2 (G-G’) and n(HE) = 0–6, without consideration of tandem products, the number of possible isomers is 1, 6, 15, 20, 15, 6, and 1, respectively. For MC2-MeOEt and n(HE) = 0 and 6, single signals are observed for both types of derivatives. At n(HE) = 1, six isomers are possible. Here, two signals, one with a shoulder, are observed for the ABA-COS (Fig. S5). However, assignment to the six isomer portions is not possible, not even for n(HE) = 5 with also six isomers and three separated signals of COS-red and — although at lower resolution — COS-ABA as well.

**Fig. 8:**
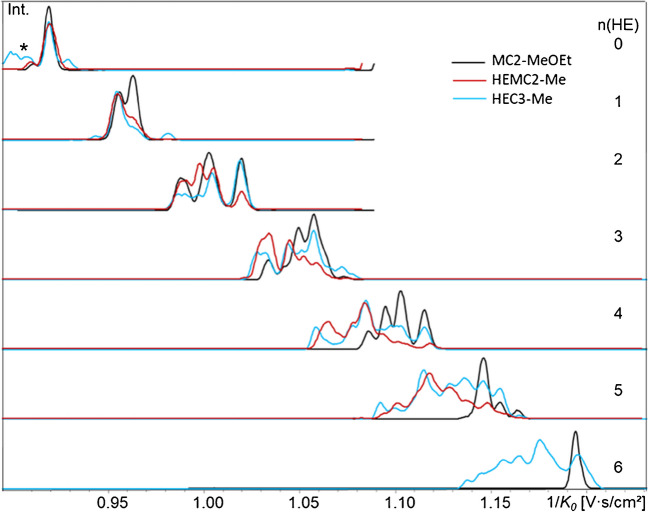
EIM of COS-red, DP2, from different HE sample types, MC-MeOEt (DS_HE_ = MDS_HE_ = 1.04), permethylated HEMC2 (MDS_HE_ 0.35), permethylated HEC3 (MDS 3.35), n(HE) = 0–6, *m/z* 433–697. [M + Na]^+^, ESI-tims-ToF–MS, positive, ultra-mode, syringe infusion. For HEMC2, the concentration of n(HE) = 5 is already too low. For HEC3, up to n(HE) = 7 is detected (not shown). Please note that each EIM is normalized to the most abundant signal; thus, intensities can neither be compared within nor among samples but only the relative intensities of a particular profile. * noise

By propagation of HE groups, the diversity of COS isomers further increases. Figure 8 shows the IM of DP2 from reductive depolymerization of MC-MeOEt (MDS = DS, inverse regioselectivity), HEMC2 (MDS 0.35), and HEC3 (MDS 3.35) for the isomer fractions with n(HE) = 0–6. With increasing MDS, the mobility range covered by an isomer fraction first increases, and above n(HE) = 6, the differences level, indicated by decreasing width of profiles in the IM (not shown). From the comparison of the three sample types, it is obvious that tandem products tend to have lower 1/*K*_*0*_, i.e., lower CCS, compared to core-substituted products, as has been observed for DP1. In the ESM, comparisons of DP2-red of the three high-MDS HECs (Fig. S6) and the three low-MDS HEMCs (Fig. S7) are displayed, showing significant differences.

## Conclusion and outlook

In conclusion, studies of the glucose derivatives (DP1) derived from three sets of samples, MC-MeOEt, HEMC, and HEC, by ESI-tims-ToF–MS have shown that the best qualitative and quantitative information regarding the HE substitution pattern is obtained for the reductively aminated (*m*ABA) glucose ethers. The separation of α- and β-anomers of glucose in IMS interferes with pattern assignment. 1,5-Anhydro-glucitols, obtained by reductive depolymerization of the permethylated HECs, showed only one peak per compound which could be assigned according to the HE position. However, in contrast to ABA derivative, quantification of regioisomers was not possible since the extent of sodiation and thus ion yield is sensitive to the methoxyethyl position in these monomeric compounds, resulting in bias. By reductive amination, anomer separation is also disabled, and in addition, due to the charge location at the tag, the position of substitution does no longer have an impact on ion yield. Thus, quantification of the isomer ratios became possible, and results were in good agreement with reference data from GLC-FID of corresponding alditol acetates. Although overall quantification over the entire *m/z* range cannot be applied, discrimination due to mass fractionation effects seems to be moderate.

When HE propagates to oligoether chains (tandem substitution products), mixtures become too complex for full separation, but at least for n(HE) = 2, the signal containing the position of the most relevant 2,2- and 6,6-HE-glucoses could be assigned. Among the compounds belonging to n(HE) = 3, the 2,3,6-HE-glc showed the highest 1/*K*_*0*_ corresponding to the largest CCS, while all tandem products (nine isomers) are “smaller.” Due to similar regioselectivity of oxirane addition, the three HEMC and three HECs showed large overall similarities but also significant qualitative and quantitative differences.

From n(HE) = 4 on, the ion mobilograms of DP1 can be considered as fingerprints of the materials they represent. Perspectively, they could provide valuable information if a pool of sample data would be established and connected to reference data and product properties. Comparison of three HECs showed a relative increase of the “smaller” isomers (i.e., the tandem products) with increasing MDS. Up to n(HE) = 10 was detected for HEC3 (MDS 3.35).

Number of substituent distribution in COS ethers is an established method to determine any deviations from randomness of substitution in and over the polysaccharide chains. By the application of IMS to COS, this information is enhanced by fingerprints for each isobaric group. Systematic collection of such data accompanied by more tedious reference analysis might open an improved sample characterization by a relatively simple and fast procedure. For COS-fingerprints of DP2, the 1,5-anhydro-glucitol-terminated COS showed improved separation and are thus the best choice for sample comparison.

With this first application of trapped ion mobility MS to cellulose ethers, the potential of this method should be outlined. Its capability was illustrated on the example of complex hydroxyethyl celluloses including low and high tandem substitution. Three types of COS-derivatives, including positive and negative mode, have been tested and compared. However, there are many further options, for instance, reduction to glucitol-terminated COS, variation of the charged tag in reductive amination, for instance, the preparation of positively charged analytes by using procainamide/4-amino-*N*-[2-diethylamino]ethyl-benzamide [38], the introduction of a quaternary ammonium tag by Girard T, or the use of other coordination metal salts. Derivatives with polar substituents can be expected to have a stronger impact of the position on the interaction of the analyte ion with the drift gas. And resolution of ion mobility can be further enhanced, although it cannot be expected to resolve all constituents due to the very large number of possible patterns in hydroxyalkyl ethers, but the application of artificial intelligence (AI) might enhance the diagnostic potential of the IM distribution patterns recorded. Coupling MS to LC would introduce an additional dimension of separation. Finally, combination with CID or other fragmentation techniques can support the identification of the substituent pattern by additional structural information during method development.

### Supplementary information

Below is the link to the electronic supplementary material.

Supplementary file1 (DOCX 916 KB)
