# Supplementary material for: Potential of ion mobility mass spectrometry in cellulose ether analysis: substitution pattern of hydroxyethyl celluloses
Source: Anal Bioanal Chem. 2024 Mar 4;416(10):2527–39. doi: 10.1007/s00216-024-05224-w (PMC10972972; doi:10.1007/s00216-024-05224-w)
Supplement: Supplementary file 1 — Supplementary file1 (DOCX 916 KB) [file 216_2024_5224_MOESM1_ESM.docx]

**Electronic Supplement Material**

**Potential of ion mobility mass spectrometry in cellulose ether analysis: Substitution pattern of hydroxyethyl celluloses**

Petra Mischnick*, Sarah Schleicher

Institute of Food Chemistry, Technische Universität Braunschweig, Schleinitzstr. 20,

38106 Braunschweig, Germany

*Corresponding authors: Petra Mischnick, p.mischnick@tu-braunschweig.de

ORCID, Petra Mischnick: 0000-0002-8313-3313

ORCID, Sarah Schleicher: 0000-0003-0159-5141

**1. Ion mobilities of homologous α,β-COS standard compounds of uniformly substituted Glc units**

ESI-tims-ToF-MS, detect-mode, syringe pump infusion, [M+Na]^+^

A: 2,3,6-tri-*O*-methyl-Glc (blue)

B: 2,3-di-*O*-methyl-6-*O*-methoxyethyl-Glc (orange)

C: 2,3-di-*O*-methoxyethyl-6-*O*-methyl-Glc (gray)

D: 2,3,6-tri-*O*-methoxyethyl-Glc (black)

As an example of the standard deviation (SD) of measurements, data for 2,3,6-Me-α,β-COS are summarized in the following table. The separation factor for DP1 has been added in order to illustrate that the relative deviation is significant smaller than that of absolute mobility values.

**
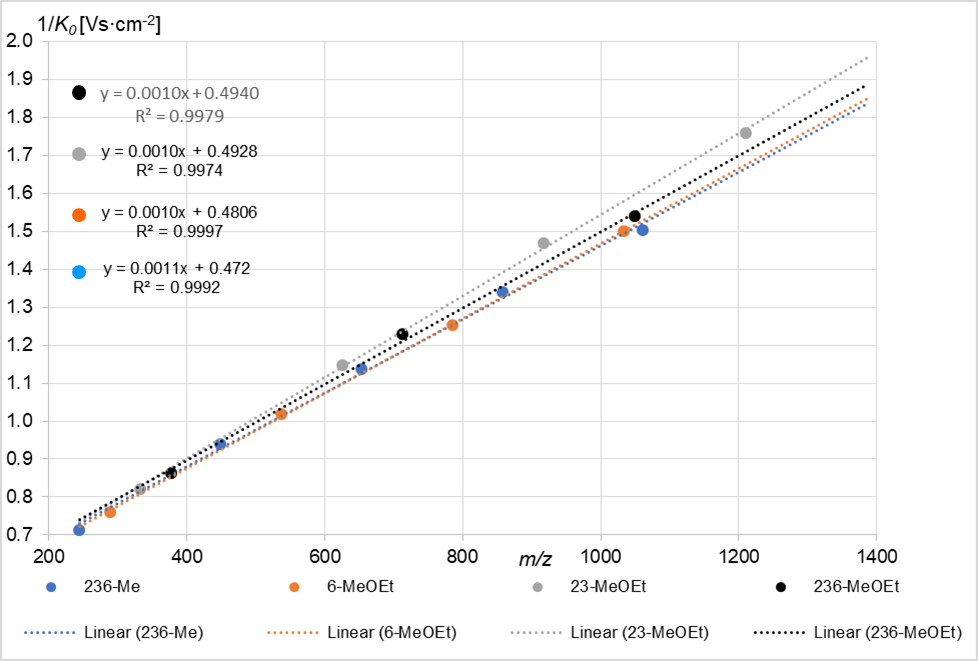
**

**Fig. S1a** Reciprocal reduced ion mobility 1/*K_0_* in VS∙cm^-2^ of homologous α,β-COS (DP1-5), [M+Na]^+^, with increasing number of MeOEt/Glc in defined positions. n(MeOEt)+n(Me)/Glc=3. ESI-tims-ToF-MS, positive, detect mode, syringe infusion. Average values of α- and β-anomers are shown if separated; in detect mode, not all α- and β-COS were separated. Linear fits are shown


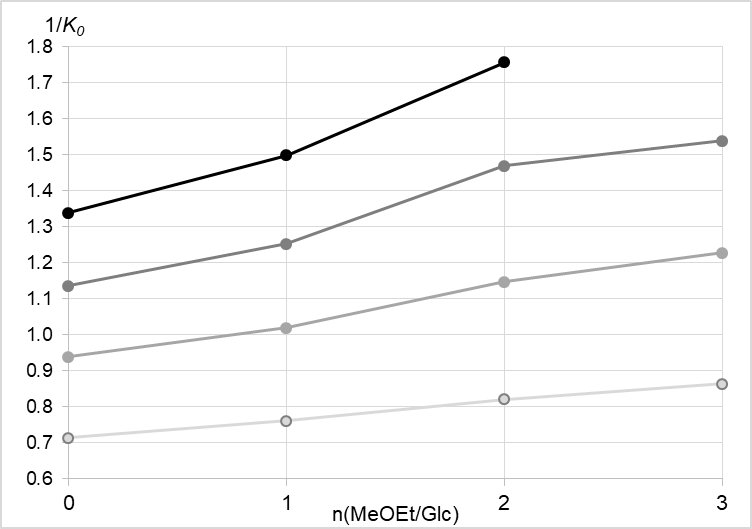


**Fig. 1b** Average ion mobilities 1/*K_0_* (VS∙cm^-2^) in dependence on the number and position of methoxyethyl groups per glucose unit in homologous α,β-COS A, B, C, and D (see above), DP1-4. n=1: 6-MeOEt, n=2: 2,3-MeOEt

From Fig. S1a, where the reciprocal ion mobilities 1/*K_0_* were referred to *m/z*, and also from Fig. S1b, it is visible, that the uniformly 2,3-*O*-methoxyethyl-6-*O*-methyl-α,β-COS (C) show a stronger increase of 1/*K_0_* than expected for their *m/z*, corresponding to larger CCS, i.e. stronger interaction with the drift gas nitrogen than expected. This is probably due to the preferred coordination of sodium involving the adjacent 2- and 3-methoxyethyl groups which might cause a more extended shape. From cellulose ether samples, it is deduced that for n(MeOEt)=1, 6-*O*-MeOEt shows the lowest CCS. For n=2, 2,6- and 3,6-di-*O*-MeOEt and tandem-substituted isomers (DP1/n(HE)=2) elute at lower 1/*K_0_* (CCS respectively), while 2,3-di-*O*-MeOEt isomers are the “biggest”. 2,3,6-Tri-*O*-methyl- (A) and 2,3-di-*O*-methyl-6-*O*-methoxyethyl-α,β-COS (B) behave equally as visible from Fig. S1a.

For the 2,3,6-tri-*O*-methoxyethyl-α,β-COS (D), only DP1-3 could be measured in the IM-window 0.6-1.8. The standard deviation for 1/ *K_0_* of A and D was ± 0.30% (A, n=6) and ± 0.27-0.61% (D series, n=3). Deviation between particular runs was systematic.

**2. Ion mobilograms of 1,5-anhydroglucitol derivatives of HEMC (COS-red, DP1)**

**
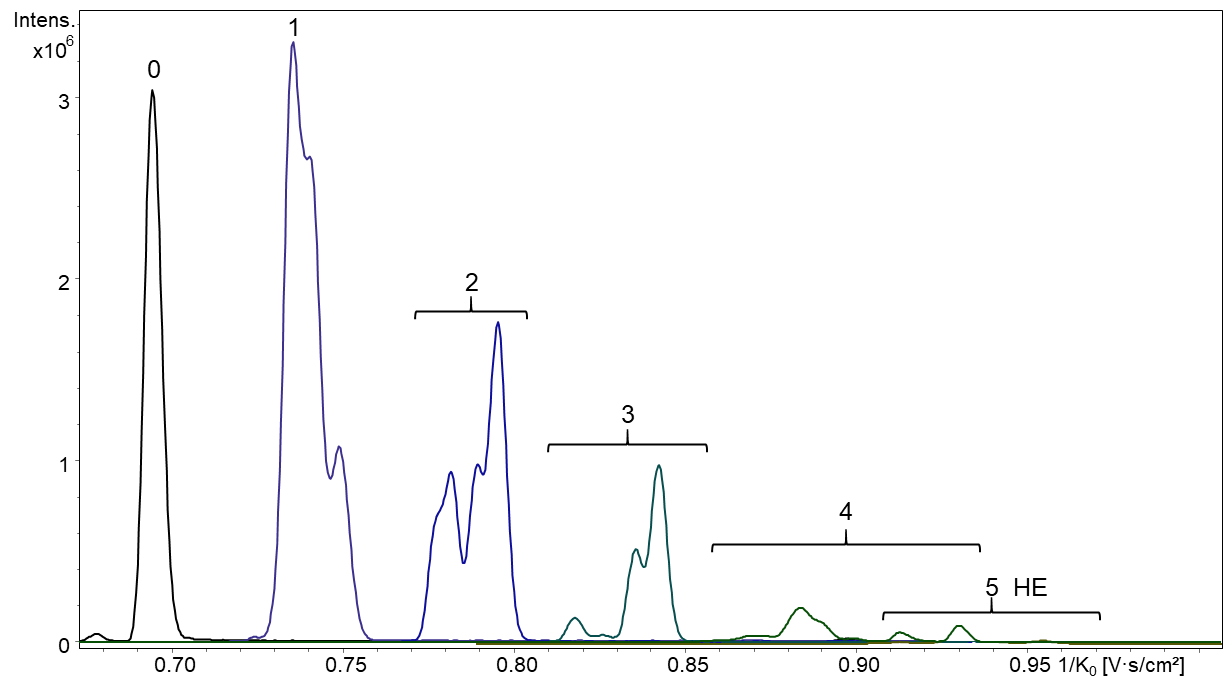
**

**Fig. S2** Overlaid EIM of HEMC2-Me (MS 0.35, [9]), COS-red, DP1, *m/z* 229-339, [M+Na]^+^, ESI-tims-ToF-MS, N_2_, positive, ultra-mode, syringe infusion. The EIM show the decreasing total contribution with n(HE). For the assignment of substituted positions see Fig. 3 in the body text. Method is not optimized with respect to quantifiability over the entire *m/z* range.

**3. Identification of tandem-reaction products in HEMC and HEC, COS-Red, DP1**


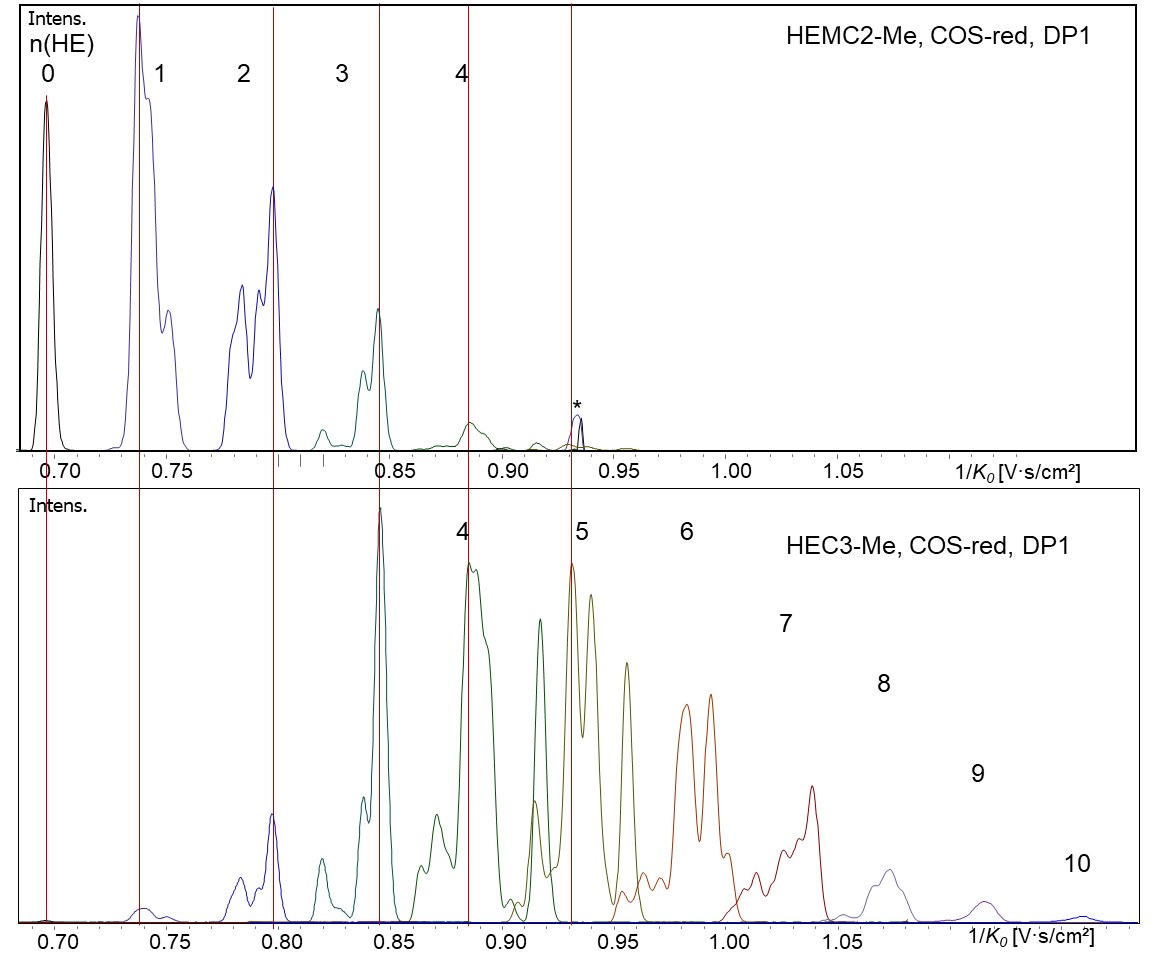


**Fig. S3** Overlaid EIM of HEMC2-Me (top, MS 0.35, *m/z* 229-449), and HEC3-Me (bottom, MDS 3.35, *m/z* 229-669). COS-red, DP1 (1,5-anhydroglucitols), [M+Na]^+^, ESI-tims-ToF-MS, N_2_, positive, ultra-mode, syringe infusion. Maximum area of HEMC2 at n(HE)=1, for HEC3 at n(HE)=3-5. The signal for n(HE)=0 is discriminated due to poorer Na-complexation ability compared to MeOEt. It is by far the most abundant portion in HEMC2. Also the other signal intensities do not directly reflect the molar portions, but allow a relative comparison of the two samples with different MS. Maximum signals of a group of isomers of a particular n(HE) are connected by red lines. *artefacts from *m/z* 229 (n(HE)=0) and *m/z* 273 EIM (n(HE)=1). Method is not optimized with respect to quantifiability over the entire *m/z* range.

**4. Comparison of a set of HEC, COS-ABA, DP1**

**
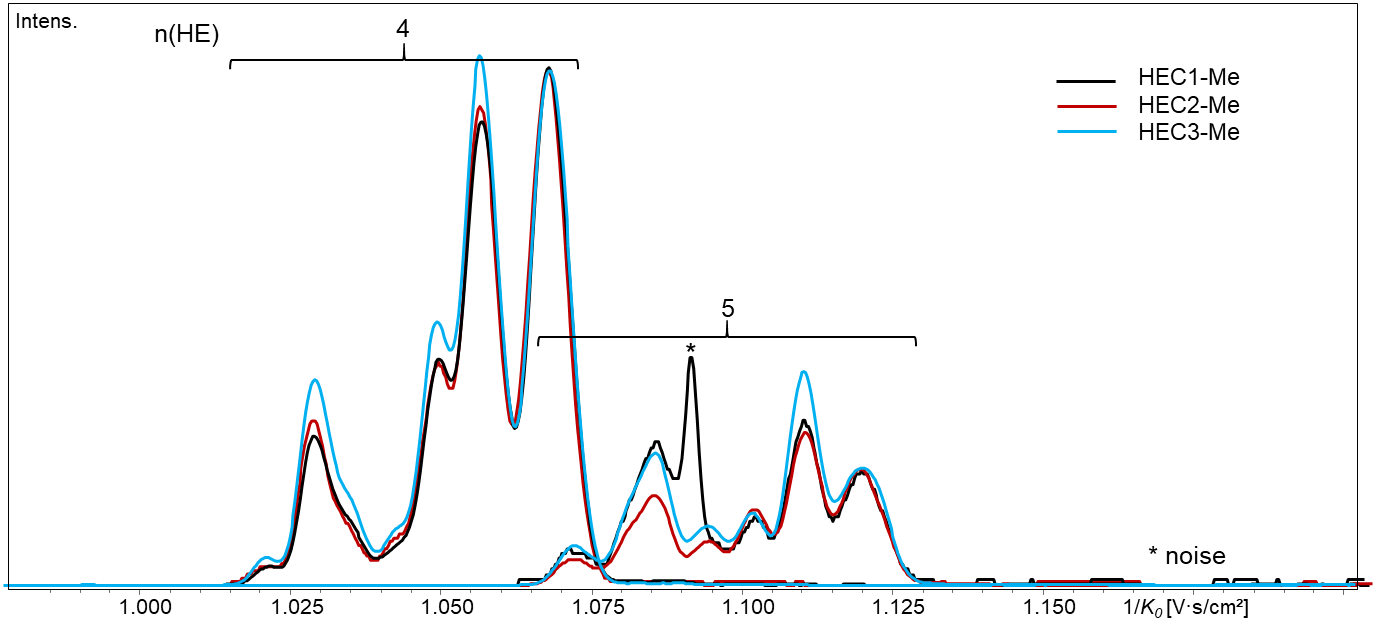
**

**
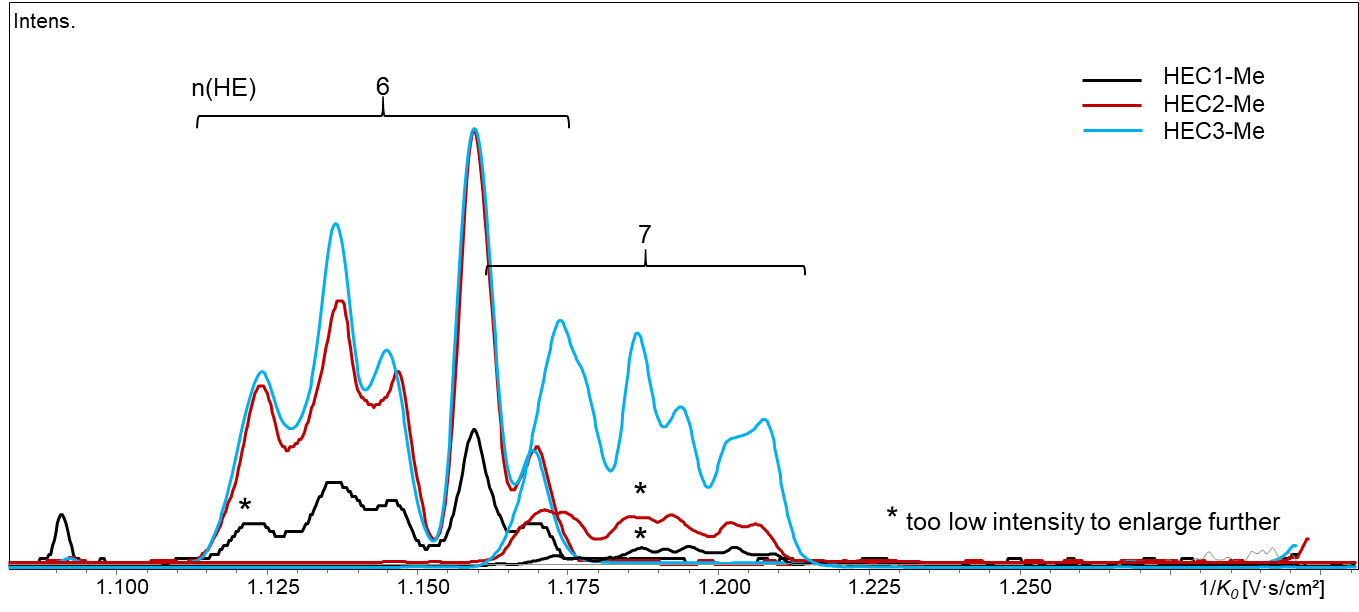
**

**
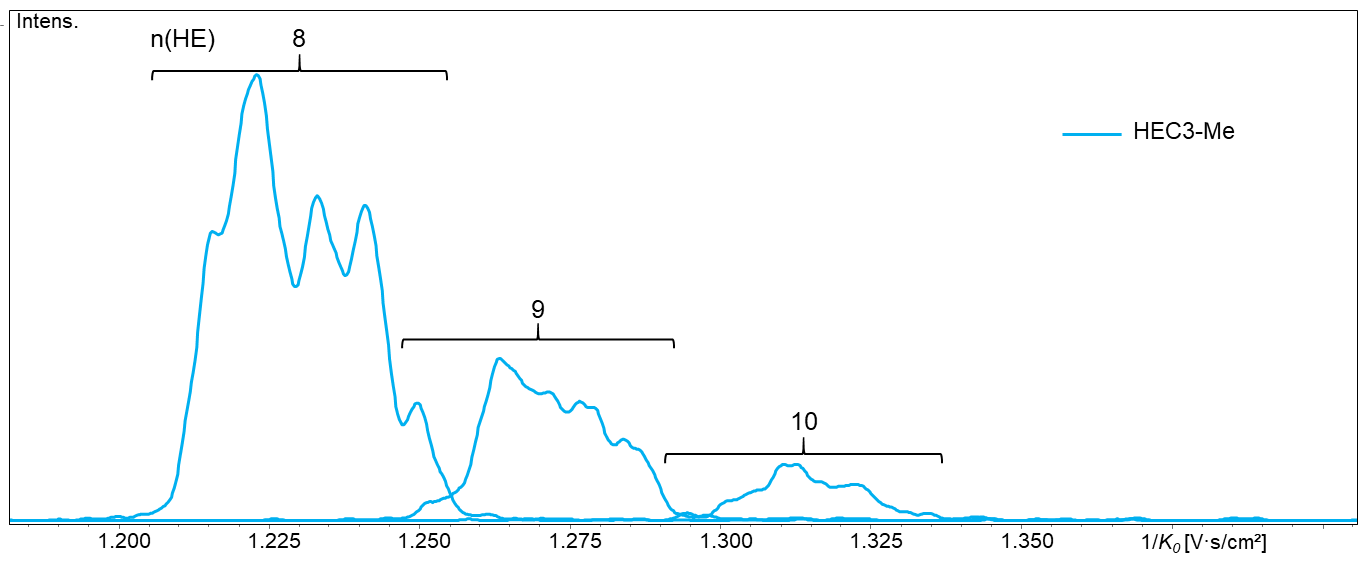
**

**Fig. S4a** Comparison of EIM of HEC1-3 (MDS 1.85, 2.06, 3.35, respectively), Me, COS-ABA, DP1, n(HE) 4‑10, *m/z* 342-782, Δ *m/z* 4. ESI-tims-ToF-MS, [M-H]^-^, negative, ultra mode, syringe infusion. EIM of n(HE)=1-3 are shown in the body text, Fig. 7. Signal intensities of the three HEC of a particular EIM were adapted for comparison.


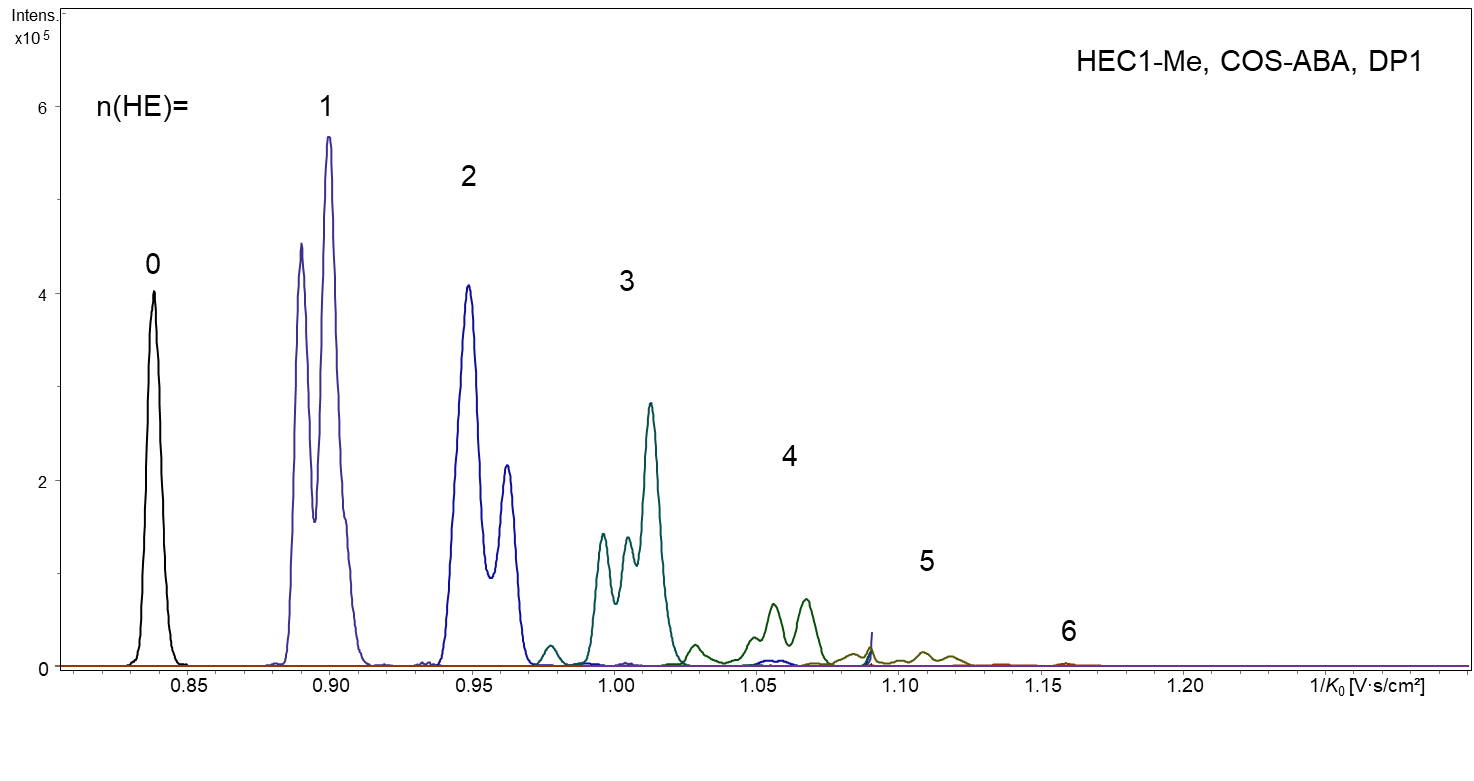


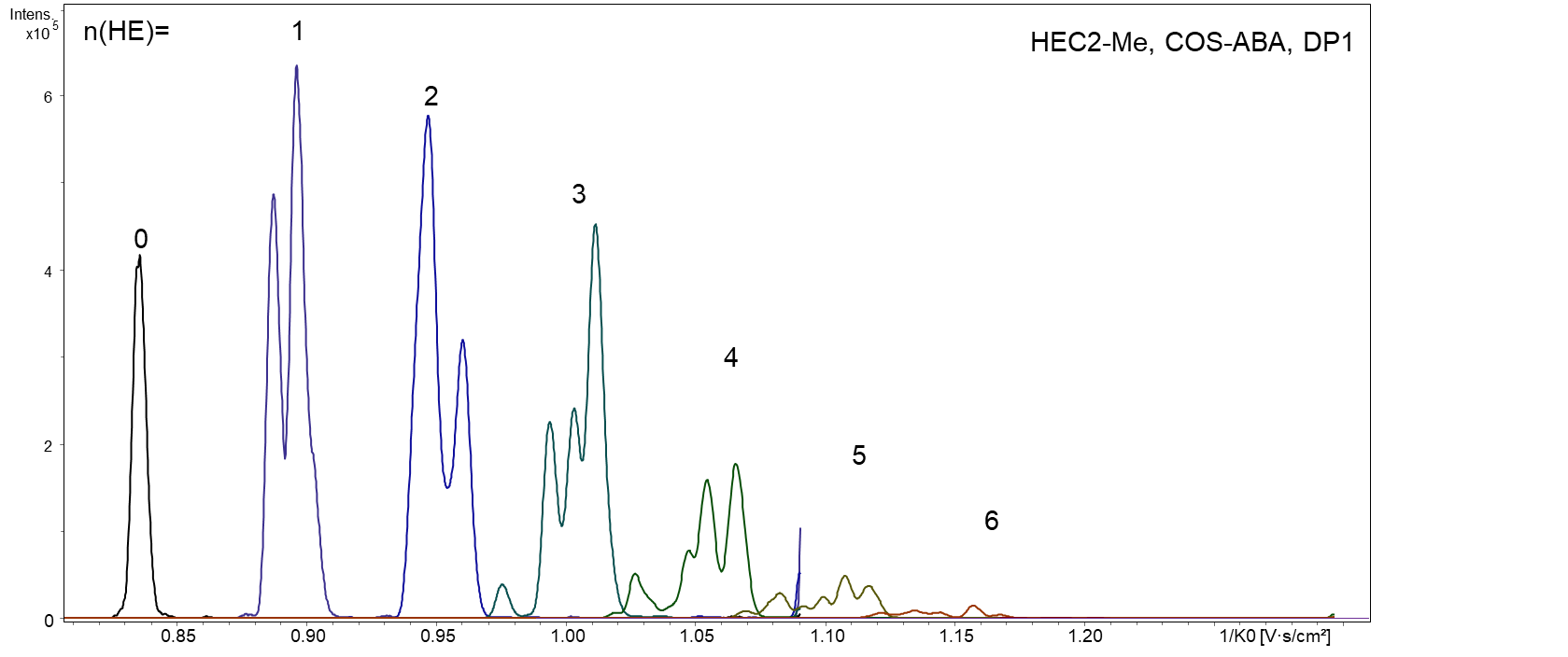


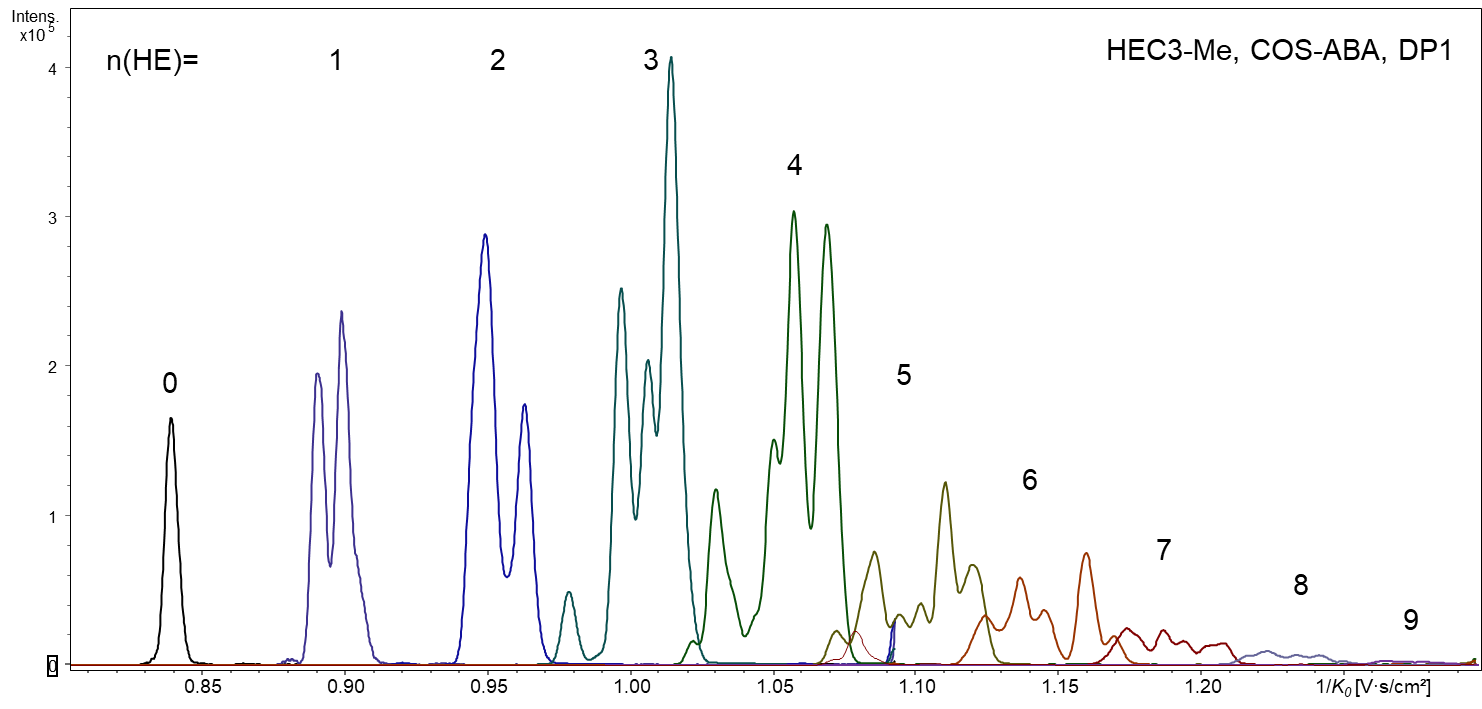


**Fig. S4b** Overlaid EIM of HEC1-3 (Me, COS-ABA, DP1), n(HE)=0-9, *m/z* 342 – 738, Δ *m/z* 44, ESI-tims-ToF-MS, [M‑H]^-^, negative, ultra mode, syringe infusion. For assignment of HE positions see Figs. 5 and 6 in the body text. For a list of EIM with enlarged intensity, see Fig. 7 in the body-text and Fig. S4a.

**Table S1** Distribution (Mol%) of n(HE) in DP1 of HEC1-3 as determined by GLC [6] and estimated from EIM peak areas from ESI-tims-ToF-MS of ABA-glucitols

| n(HE) | HEC1  GLC | HEC1  IM-MS | HEC2  GLC | HEC2  IM-MS | HEC3  GLC | HEC3  IM-MS |
| --- | --- | --- | --- | --- | --- | --- |
| 0 | 16.5 | 11.8 | 14.3 | 8.8 | 5.8 | 3.6 |
| 1 | 26.7 | 33.2 | 25.4 | 25.6 | 12.2 | 10.6 |
| 2 | 26.0 | 26.3 | 28.2 | 26.9 | 16.0 | 15.4 |
| 3 | 16.4 | 19.2 | 20.1 | 21.9 | 29.9 | 24.3 |
| 4 | 11.2 | 7.0 | 8.7 | 11.4 | 17.0 | 25.1 |
| 5 | 3.0 | 1.9 | 3.0 | 3.9 | 14.0 | 10.6 |
| 6 | 0.3 | 0.4 | 0.3 | 1.0 | 4.9 | 6.6 |
| 7 | n.d. | 0.1 | n.d. | 0.3 | 0.2 | 2.7 |
| 8 |  | 0.1 |  | 0.1 | n.d. | 9.8 |
| 9 |  |  |  |  |  | 0,3 |
| 10 |  |  |  |  |  | 0.1 |
| MDS | 1.89 | 1.85 | 1.94 | 2.19 | 3.03 | 3.26 |
| MDS_Zeisel_ | 1.85 | 1.85 | 2.06 | 2.06 | 3.35 | 3.35 |

**5. Fingerprints of COS-red and COS-ABA, DP2**

The ion mobilograms (ESI-tims-ToF-MS, ultra-mode, syringe infusion) of DP2 can be used as a fingerprint of the HE cellulose ethers. The following figures show a comparison of methoxy­ethylated MC: 1,5-Anhydroalditol-terminated (COS-red) and *m*ABA-labeled patterns (COS-ABA).


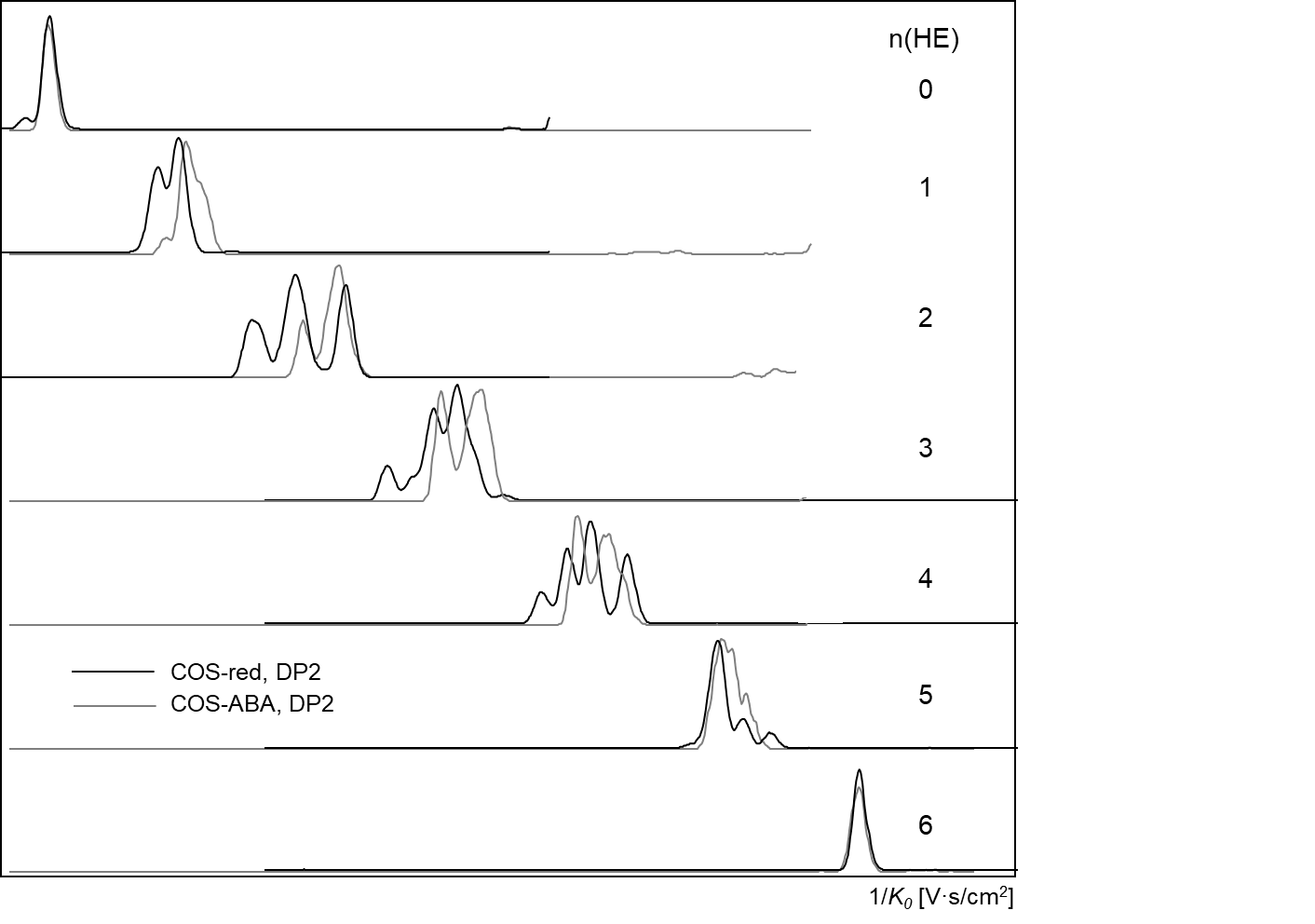


**Fig. S5** MC2-MeOEt (DS_MeOEt_ 1.04), no-tandem products. EIM of DP2, n(HE)=0 to n(HE)=6 are shown. ESI-tims-ToF-MS, ultra mode, syringe infusion. Black: COS-red, [M+Na]^+^, *m/z* range 433 – 697. 1/*K_0_* range: 0.9186 – 1.1976. Gray: COS-ABA, [M‑H]^-^, *m/z* range 546 – 810. 1/*K_0_* range: 1.0364 – 1.3154. EIM are aligned for direct comparison. Both derivatives show the same slope of 1/*K_0_* with *m/z*, thus, the IM of ABA-COS was shifted by +0.118 against COS-red.


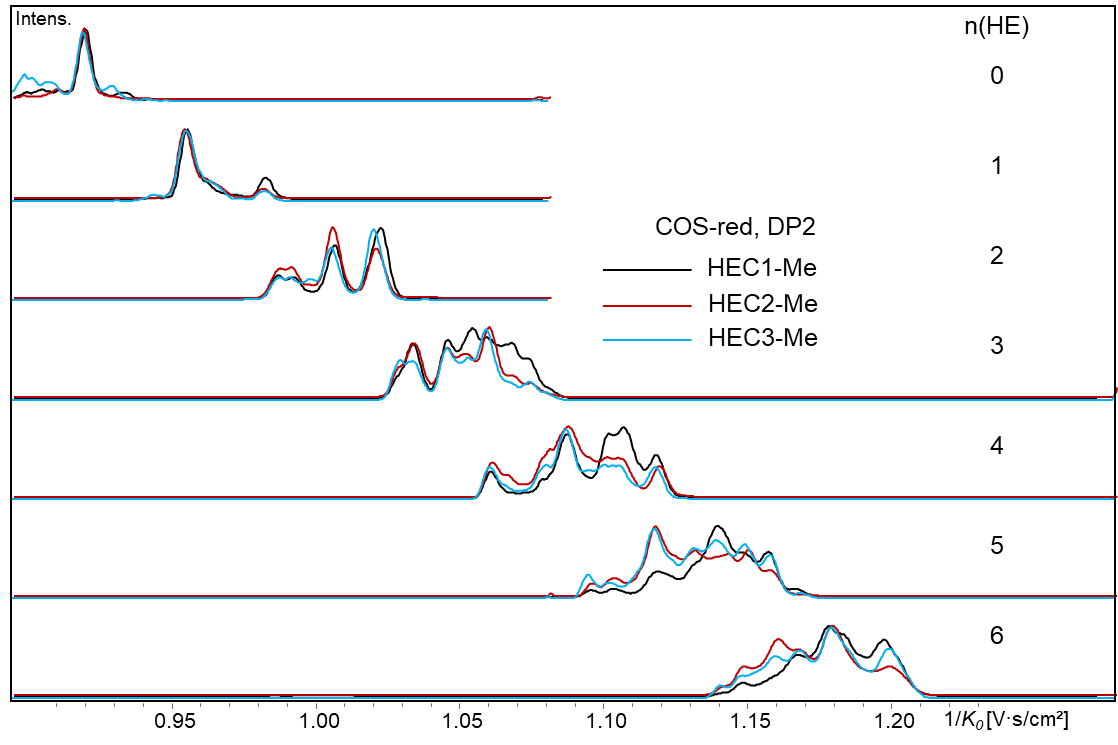


**Fig. S6**. EIM of DP2 of HEC1-3, COS-red; n(HE)=0-6, *m/z* 433-697, [M+Na]^+^, positive mode, ESI-tims-ToF-MS, ultra mode, syringe infusion. HEC1: MDS 1.85, HEC2: MDS 2.06, HEC3: MDS 3.35.Iintensities of the EIM are not directly comparable, since each mobilogram is normalized to the maximum peak. Due to the large MS values, concentration of n(HE)=0 is the lowest and increases with n.

Fig. S6 compares the IM of DP2 of HEC1-3 (COS-red). Complexity of isomer fractions continuously increases with n(HE) due to the tandem reaction products. The IM profiles of HEC1 and HEC2 with similar MS significantly differ. For DP1 tandem products had been found to show lower 1/*K_0_* than core-substituted isomers. Comparison of HEC with tandem-free MC-MeOEt (Fig. S5, top) shows that the 236-2’3’6’-substituted DP2 is again located at the right border of the n(HE)=6 profile, at about 1/*K_0_* 1.2, supporting the assumption that this tendency also fits for DP2.


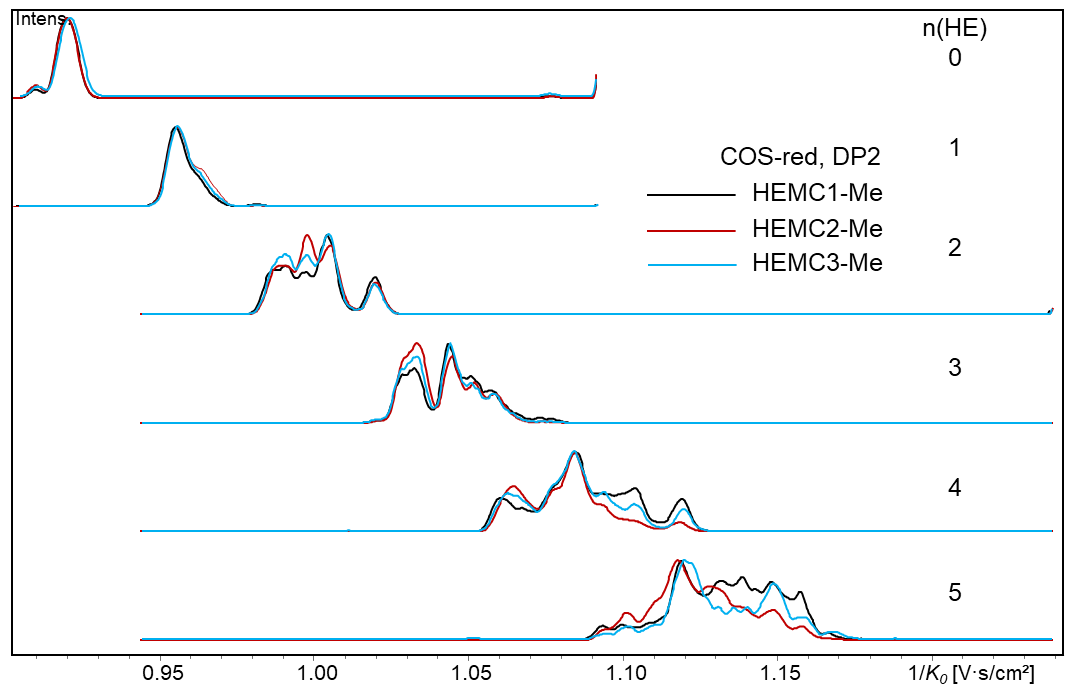


**Fig. S7** EIM of DP2 of HEMC1-3, COS-red; n(HE)=0-5, *m/z* 433-653, [M+Na]^+^, positive mode, ESI-tims-Tof-MS, ultra mode, syringe infusion. Complexity of isomer fractions continuously increases with n(HE) due to tandem reaction products. HEMC1: MDS 0.21, HEMC2: MDS 0.35, HEMC3: MDS 0.17. Intensities are not directly comparable, since each EIM is normalized to the maximum peak. Due to the low MDS-values, n=0 strongly dominates and intensities fast decrease with n(HE); thus n=4 and 5 are smoothed and might contain some noise; compare Fig. S6 of HECs-with high MS.

In Fig. S7 the corresponding comparison of the set of HEMCs, COS-red, DP2, also shows significant differences of the IM-fingerprint.

**References**

Citations refer to the reference list in the body text.
